# Stability-Diversity Tradeoffs Impose Fundamental Constraints on Selection of Synthetic Human V_H_/V_L_ Single-Domain Antibodies from *In Vitro* Display Libraries

**DOI:** 10.3389/fimmu.2017.01759

**Published:** 2017-12-12

**Authors:** Kevin A. Henry, Dae Young Kim, Hiba Kandalaft, Michael J. Lowden, Qingling Yang, Joseph D. Schrag, Greg Hussack, C. Roger MacKenzie, Jamshid Tanha

**Affiliations:** ^1^Human Health Therapeutics Research Centre, National Research Council Canada, Ottawa, ON, Canada; ^2^Human Health Therapeutics Research Centre, National Research Council Canada, Montréal, QC, Canada; ^3^School of Environmental Sciences, University of Guelph, Guelph, ON, Canada; ^4^Department of Biochemistry, Microbiology and Immunology, University of Ottawa, Ottawa, ON, Canada

**Keywords:** single-domain antibody, synthetic antibody, human V_H_/V_L_, phage display, protein engineering

## Abstract

Human autonomous V_H_/V_L_ single-domain antibodies (sdAbs) are attractive therapeutic molecules, but often suffer from suboptimal stability, solubility and affinity for cognate antigens. Most commonly, human sdAbs have been isolated from *in vitro* display libraries constructed *via* synthetic randomization of rearranged V_H_/V_L_ domains. Here, we describe the design and characterization of three novel human V_H_/V_L_ sdAb libraries through a process of: (i) exhaustive biophysical characterization of 20 potential V_H_/V_L_ sdAb library scaffolds, including assessment of expression yield, aggregation resistance, thermostability and tolerance to complementarity-determining region (CDR) substitutions; (ii) *in vitro* randomization of the CDRs of three V_H_/V_L_ sdAb scaffolds, with tailored amino acid representation designed to promote solubility and expressibility; and (iii) systematic benchmarking of the three V_H_/V_L_ libraries by panning against five model antigens. We isolated ≥1 antigen-specific human sdAb against four of five targets (13 V_H_s and 7 V_L_s in total); these were predominantly monomeric, had antigen-binding affinities ranging from 5 nM to 12 µM (average: 2–3 µM), but had highly variable expression yields (range: 0.1–19 mg/L). Despite our efforts to identify the most stable V_H_/V_L_ scaffolds, selection of antigen-specific binders from these libraries was unpredictable (overall success rate for all library-target screens: ~53%) with a high attrition rate of sdAbs exhibiting false positive binding by ELISA. By analyzing V_H_/V_L_ sdAb library sequence composition following selection for monomeric antibody expression (binding to protein A/L followed by amplification in bacterial cells), we found that some V_H_/V_L_ sdAbs had marked growth advantages over others, and that the amino acid composition of the CDRs of this set of sdAbs was dramatically restricted (bias toward Asp and His and away from aromatic and hydrophobic residues). Thus, CDR sequence clearly dramatically impacts the stability of human autonomous V_H_/V_L_ immunoglobulin domain folds, and sequence-stability tradeoffs must be taken into account during the design of such libraries.

## Introduction

The concept of an autonomous single immunoglobulin variable domain (single-domain antibodies or sdAbs) as the smallest representation of an antigen-binding-competent antibody was first described by Ward et al. in the mouse (1). With the discovery of naturally occurring heavy chain-only antibodies in *Camelidae* (2) and in cartilaginous sharks (3) several years later (the single variable domains of which can recognize antigen autonomously), it became clear that sdAbs represented not only a theoretical possibility but a viable immunological solution to the problem of antigen recognition. Although the human humoral immune system produces only conventional antibodies with paired heavy and light chains and not sdAbs, the question of whether human sdAbs (autonomous variable heavy- or light-chain domains, V_H_s or V_L_s) could be isolated and/or molecularly engineered *in vitro* was brought to light.

The identification, engineering and biophysical characterization of a handful of non-antigen-specific human V_H_/V_L_ sdAbs has been extensively reported and discussed (4). The first efforts to produce human V_H_/V_L_ sdAbs with novel antigen-binding specificities used “camelized” scaffolds that incorporated the solubilizing framework region (FR) substitutions found in camelid sdAbs (5–9). Although this approach yielded antigen-specific sdAbs with excellent solubility and biophysical properties, it relied on undesirable sequence deviation from the human IGHV germline. Later, rare fully human rearranged V_H_ and V_L_ variable domains were discovered that were autonomously stable and monomeric and large phage display libraries were constructed by randomizing their complementarity-determining regions (CDRs), although it was clear from the mid-2000s that certain CDR sequences (potentially low in hydrophobic content and rich in negative charge) were better compatible with solubility and stability of these molecules (9–11). There are now many examples of fully human antibodies (primarily V_H_s) isolated from such libraries against a variety of targets, including α-amylase (12), β-galactosidase (13, 14), *Candida albicans* MP65 and SAP-2 (15), carbonic anhydrase (12), CD154 (16), CD28 (17), CD40 (18, 19), CD40L (20), *Clostridium difficile* toxin B (21), EGFR (22), glypican-2 (23), glypican-3 (24), human serum albumin (HSA) (25–27), lysozyme (28–30), maltose-binding protein (31), MDM4 (32), mesothelin (33), TNF-α (34), TNFR1 (35), and VEGF (22). These fully human V_H_/V_L_ sdAbs exhibit a variety of antigen-binding modes and functional activities and several have entered clinical development, where they have been generally well-tolerated albeit unexpectedly immunogenic (36, 37).

Here, we report the design, construction and characterization of three novel phage-displayed, synthetically randomized human V_H_/V_L_ sdAb libraries. We attempted to circumvent the unfavorable biophysical properties of many human V_H_/V_L_ sdAbs by (i) selecting ultra-stable V_H_/V_L_ sdAbs tolerant to CDR modification as library scaffolds, (ii) maximizing randomized sequence diversity in CDRs using trinucleotide mutagenesis, and (iii) spiking the library with negatively charged residues to encourage solubility. Similarly to the experiences of others, we were able to isolate monomeric, high-affinity V_H_/V_L_ sdAbs from the libraries against some antigens but not against others. The stochastic process of selecting binders from human V_H_/V_L_ sdAb libraries is likely a consequence of fundamental tradeoffs between CDR sequence and human V_H_/V_L_ sdAb stability and aggregation resistance.

## Materials and Methods

### Identification of Human Autonomous V_H_/V_L_ sdAb Scaffolds

The human autonomous V_H_ and V_L_ sdAb scaffolds used in this study (Table 1; Figure S1 in Supplementary Material) were isolated as previously described by To et al. (38) and Kim et al. (39). Disulfide-stabilized versions of each V_H_/V_L_ sdAb (bearing an intradomain disulfide linkage formed between Cys residues at IMGT positions 54 and 78) were produced by overlap extension PCR as described in Kim et al. (40).

**Table 1:** Properties of human V_H_ and V_L_ single-domain antibody scaffolds used in this study.

Type	Scaffold^a^	Disulfide linkages^b^	Germline rearrangement	CDR3 length (aa)	*T*_m_ (°C)	Monomer (%)	Reference
V_L_	VL383	23–104	IGKV3-20–IGKJ2	9	57.3	>95	(39)
	VL383_SS_	23–104, 54–78	IGKV3-20–IGKJ2	9	73.7	85.3	
	VL382	23–104	IGKV3-20–IGKJ1	9	70.1	>95	(39)
	VL382_SS_	23–104, 54–78	IGKV3-20–IGKJ1	9	83.3	>95	
	VL335	23–104	IGKV3-20–IGKJ1	9	61.7	>95	(39)
	VL335_SS_	23–104, 54–78	IGKV3-20–IGKJ1	9	79.0	>95	(39)
	VL330	23–104	IGKV1-39–IGKJ2	9	62.8	>95	(39)
	VL330_SS_	23–104, 54–78	IGKV1-39–IGKJ2	9	83.7	>95	
	VL325	23–104	IGKV3-11–IGKJ4	9	68.5	>95	(39)
	VL325_SS_	23–104, 54–78	IGKV3-11–IGKJ4	9	82.5	>95	(39)
V_H_	VH420	23–104	IGHV3-15–IGHJ4	10	57.8	>95	(38)
	VH420_SS_	23–104, 54–78	IGHV3-15–IGHJ4	10	67.3	>95	
	VH428	23–104	IGHV3-49–IGHJ4	14	62.3	>95	(38)
	VH428_SS_	23–104, 54–78	IGHV3-49–IGHJ4	14	73.1	>95	
	VH429	23–104	IGHV3-23–IGHJ4	12	58.5	>95	(38)
	VH429_SS_	23–104, 54–78	IGHV3-23–IGHJ4	12	71.8	>95	
	VHB82	23–104	IGHV3-23–IGHJ6	6	57.9	>95	(38)
	VHB82_SS_	23–104, 54–78	IGHV3-23–IGHJ6	6	72.9	80.8	
	VHM81	23–104	IGHV3-23–IGHJ3	14	66.9	>95	(38)
	VHM81_SS_	23–104, 54–78	IGHV3-23–IGHJ3	14	76.8	>95	

*^a^Full-length amino acid sequences are listed in Figure S1 in Supplementary Material*.

*^b^IMGT numbering*.

### CDR Shuffling

All three CDRs of each V_H_/V_L_ scaffold were simultaneously exchanged for those listed in Table 2 (20 V_H_/V_L_ scaffolds × 12 CDR sets = 240 CDR-shuffled variants). DNA constructs encoding the CDR-shuffled variants were synthesized commercially (GeneArt/Life Technologies, Regensberg, Germany) and subcloned into the pSJF2H bacterial expression vector (12).

**Table 2:** Amino acid sequences of complementarity-determining region (CDR) sets introduced into human V_H_/V_L_ single-domain antibody (sdAb) scaffolds for biophysical stability assessment.

V_L_ CDR set	CDR-L1^a^	CDR-L2^b^	CDR-L3^c^	CDR-L3 length (aa)
1	RASQSVLVHLA	GDSYRAD	QQTFYPST	8
2	RASQSVISNLA	GDSFRAF	QQVAHPTT	8
3	RASQSVTDTLLA	GISHRAD	QQLVHPFT	8
4	RASQSVVHNLA	GLSTRAH	QQFDHPYT	8
5	RASQSVSSNLA	GASLRAT	QQYLIPPAT	9
6	RASQSVAPSLLA	GTSTRAP	QQPTLFPTT	9
7	RASQSVNLPYLA	GVSTRAY	QQIAVTPYT	9
8	RASQSVDYNLA	DISFRAN	QQSLSPPAT	9
9	RASQSVNITSLA	GTSTRAD	QQTNTHHPVT	10
10	RASQSVSSYLA	GLSLRAV	QQTDSFFPFT	10
11	RASQSVPIVLA	GHSLRAD	QQLAFFDPFT	10
12	RASQSVYLNLA	GVSVRAD	QQILLFYPHT	10

**V_H_ CDR set**	**CDR-H1^d^**	**CDR-H2^e^**	**CDR-H3^f^**	**CDR-H3 length (aa)**
1	SNAWMS	RITSKTDGGTTD	DQANAFDI	8
2	DGYAMH	VTNNGGSTS	QSITGPTGAFDI	12
3	SSYAMS	AISGGGDHTY	EGMVRGVSSAPFDY	14
4	ISESMT	AISSSGGSTY	KKIDGARYDY	10
5	NTLSMG	AVSRSGGSTY	AATKSNTTAYRLSFDY	16
6	SMYRMG	VITRNGSSTY	TSGSSYLDAAHVYDY	15
7	SMDPMA	AGSSTGRTTY	APYGANWYRDEYAY	14
8	SRYPVA	VISSTGTSTY	NSQRTRLQDPNEYDY	15
9	SNRNMG	GISWGGGSTR	EFGHNIATSSDEYDY	15
10	NFYAMS	GVSRDGLTTL	VITGVWNKVDVNSRSYHY	18
11	SPTAMG	HITWSRGTTR	STFLRILPEESAYTY	15
12	DNYAMA	TIDWGDGGAR	ARQSRVNLDVARYDY	15

*^a^IMGT positions 24–40 in the acceptor sdAb were replaced with the indicated sequence*.

*^b^IMGT positions 56–69 in the acceptor sdAb were replaced with the indicated sequence*.

*^c^IMGT positions 105–117 in the acceptor sdAb were replaced with the indicated sequence*.

*^d^IMGT positions 35–40 in the acceptor sdAb were replaced with the indicated sequence*.

*^e^IMGT positions 55–66 in the acceptor sdAb were replaced with the indicated sequence*.

*^f^IMGT positions 105–117 in the acceptor sdAb were replaced with the indicated sequence*.

### Design and Construction of Synthetic Human V_H_/V_L_ sdAb Phage Display Libraries

Three phage-displayed sdAb libraries were constructed by *in vitro* randomization of the sdAb scaffolds VH428, VHB82_SS_ and VL383_SS_. Briefly, nondegenerate oligonucleotides spanning each sdAb were chemically synthesized using the phosphoramidite method (GeneArt/Life Technologies) and purified by HPLC. CDRs were randomized *via* incorporation of defined mixtures of trinucleotide phosphoramidite building blocks (41) during oligonucleotide synthesis. Oligonucleotides were assembled without amplification by Klenow fragment extension, gel purified using a GeneJET™ gel extraction kit (Thermo-Fisher, Waltham, MA, USA) according to the manufacturer’s instructions, and resuspended in a total volume of 1 mL TE buffer (10 mM Tris-HCl, 1 mM EDTA, pH 8.0). Non-amplified library DNA was quantified by real-time PCR using Fast SYBR™ Green master mix (Thermo-Fisher), and a StepOnePlus™ real-time PCR system (Thermo-Fisher), then approximately 232–1,069 ng (4.3 × 10^11^ to 1.5 × 10^12^ molecules) of DNA was PCR-amplified with Phusion^®^ high-fidelity DNA polymerase (Thermo-Fisher) using M13F and M13R primers (sequences in Table S1 in Supplementary Material), gel purified using a GeneJET™ gel extraction kit, then digested and ligated between the *Nco*I and *Not*I sites of the phagemid vector pMED1 (42). *Escherichia coli* TG1 cells were transformed with the ligation products by electroporation, yielding final library sizes between 1.0 × 10^10^ and 2.3 × 10^10^ independent transformants, ≥95% of which carried V_H_/V_L_ sdAb inserts as shown by colony PCR.

### Antibodies, Proteins and Reagents

Recombinant human CEACAM6 (residues 145–232) and mouse GITR (residues 20–153) ectodomains were produced by transient transfection of HEK293-6E cells as previously described (43). HSA was from Sigma-Aldrich (St. Louis, MO, USA; Cat. No. A9511). Recombinant human CTLA4 ectodomain (residues 1–162) was from Sino Biological (Beijing, China; Cat. No. 11159-H08H). *E. coli* 0157:H7 intimin (residues 658–934) fused C-terminally to maltose-binding protein (MBP-intimin) was from GenScript (Piscataway, NJ, USA). Horseradish peroxidase-conjugated antibodies used in ELISA (mouse anti-M13, Cat. No. 27942101; mouse anti-c-Myc, Cat. No. 11814150001; rabbit anti-6 × His, Cat. No. A190-114P) were from GE Healthcare (Piscataway, NJ, USA), Bethyl Laboratories (Montgomery, TX, USA) and Roche Diagnostics (Basel, Switzerland), respectively. M13K07 helper phage was from New England BioLabs (Ipswich, MA, USA) and M13K07ΔpIII hyper phage was from Progen Biotechnik (Heidelberg, Germany).

### Isolation of Antigen-Specific Human V_H_/V_L_ sdAbs by Panning

Phage particles displaying monovalent V_H_/V_L_ sdAbs were prepared by rescue of the three synthetic human V_H_/V_L_ sdAb phagemid libraries with M13K07 helper phage as previously described (42). Briefly, 2 × YT broth (4 L) containing 1% (w/v) glucose and 100 µg/mL ampicillin was inoculated with 1 mL phagemid-bearing *E. coli* TG1 cells (7.3 × 10^10^ to 1.6 × 10^11^) and grown at 37°C with 250 rpm shaking to an OD_600_ of 0.5. Phagemid-bearing cells were superinfected with M13K07 helper phage at a multiplicity of infection of 20:1 at 37°C for 30 min with no shaking, then pelleted and resuspended in 6 L of 2 × YT broth containing 100 µg/mL ampicillin and 50 µg/mL kanamycin. The next day, phage particles were purified from bacterial supernatants using two rounds of polyethylene glycol precipitation; neither heat treatment nor filtration steps were used to remove residual bacterial cells. For panning, 5 µg of each protein in 35 µL phosphate-buffered saline (PBS), pH 7.4, was adsorbed overnight at 4°C in wells of NUNC MaxiSorp™ 96-well microtiter plates (Thermo-Fisher). The next day, wells were blocked with 200 µL PBS containing either 2% (w/v) bovine serum albumin (BSA) or 2% (w/v) skim milk for 1 h at 37°C, then rinsed 3 × with PBS. Approximately 5 × 10^11^ infective library phage particles (5 × 10^12^ virions) were added to wells in 100 µL of PBS containing 1% BSA or skim milk and 0.1% (v/v) Tween-20 for 2 h at room temperature. The blocking protein (BSA and skim milk) was switched in alternate rounds of panning, except for panning on HSA in which only skim milk was used. Wells were washed 5 × with PBS containing 0.1% Tween-20, 2 × with PBS and then bound phage were eluted for 10 min with 50 µL of 100 mM triethylamine, neutralized with 50 µL 1 M Tris-HCl, pH 7.5, and used to reinfect exponentially growing *E. coli* TG1 cells. The cultures were superinfected with M13K07 helper phage. The next day, amplified phage were purified by polyethylene glycol precipitation from 10 mL overnight cultures and used in subsequent panning rounds. After four or five rounds of selection, antigen-specific V_H_- or V_L_-displaying phage clones were identified by their binding in polyclonal and monoclonal phage ELISA as previously described (42).

### Selection for Monomeric and Expressible Human V_H_/V_L_ sdAbs by Panning

Phage particles displaying monovalent V_H_/V_L_ sdAbs were prepared by rescue of phagemid libraries with M13K07 helper phage as described above. Phage particles displaying multivalent V_H_/V_L_ sdAbs on all copies of pIII were prepared as described above, except that: (i) smaller volumes (100 mL) of 2 × YT broth were inoculated with 1 mL phagemid-bearing *E. coli* TG1 cells, (ii) phagemid libraries were rescued with M13K07ΔpIII hyper phage at a multiplicity of infection of 10:1, and (iii) the final overnight culture volume was 150 mL of 2 × YT broth.

For panning, 5 µg of either protein A (for the VHB82_SS_ library; Thermo-Fisher) or protein L (for the VL383_SS_ library; Thermo-Fisher) in 50 µL PBS, pH 7.4, was adsorbed overnight at 4°C in wells of NUNC MaxiSorp™ 96-well microtiter plates. The next day, wells were blocked with 300 µL PBS containing either 2% BSA or skim milk for 1 h at 37°C, then rinsed 1 × with PBS. Approximately 10^11^ infective library phage particles (10^12^ virions) were added to wells in 100 µL of PBS containing 1% skim milk and 0.1% Tween-20 for 2 h at room temperature. Wells were washed 5 × with PBS containing 0.1% Tween-20, 2 × with PBS and then bound phage were eluted for 10 min with 50 µL of 100 mM triethylamine, neutralized with 50 µL 1 M Tris-HCl, pH 7.5, and used to reinfect exponentially growing *E. coli* TG1 cells. The cultures were superinfected with either M13K07 helper phage or M13K07ΔpIII hyper phage. The next day, amplified phage were purified by polyethylene glycol precipitation from 10 mL overnight cultures and used in subsequent panning rounds. After three rounds of selection, pools of sdAb-phage (either phage bound by and eluted from protein A/L, or phage amplified from overnight cultures) were interrogated using next-generation DNA sequencing (NGS) as described below.

### Soluble V_H_/V_L_ Protein Expression

Monomeric V_H_/V_L_ sdAbs bearing C-terminal 6 × His and c-Myc tags were expressed from overnight cultures of *E. coli* TG1 cells grown in 250 mL to 1 L of 2 × YT broth under IPTG (isopropyl β-D-1-thiogalactopyranoside) induction, then extracted from periplasmic space by osmotic sucrose shock and purified by immobilized metal affinity chromatography as previously described (42). For small-scale expression screening, 5 mL overnight cultures were grown as above, lysed using FastBreak™ reagent (Promega, Madison, WI, USA) and sdAbs purified using PureProteome™ nickel magnetic beads (EMD Millipore, Billerica, MA, USA). Expression yields from 5 mL cultures were determined using the Bradford protein assay (Bio-Rad, Hercules, CA, USA) as per manufacturer’s instructions with a V_H_H sdAb of known concentration as the protein standard. Titration ELISAs using soluble V_H_/V_L_ sdAbs were performed as previously described (42, 44, 45), using either anti-6 × His or anti-c-Myc secondary antibodies to detect binding.

### Size Exclusion Chromatography (SEC) and SEC with Multiangle Light Scattering (MALS)

Size exclusion chromatography analyses of monomeric V_H_/V_L_ sdAbs were conducted using a Superdex™ 75 GL column (GE Healthcare) connected to an ÄKTA FPLC protein purification system (GE Healthcare) as previously described (42). UPLC-SEC-MALS analyses of V_H_/V_L_ sdAbs were conducted essentially as previously described (39) using an Acquity BEH-125 column (Waters, Milford, MA) connected to an Acquity UPLC H-Class Bio system (Waters) with miniDAWN™ MALS detector and Optilab^®^ UT-rEX™ refractometer (Wyatt Technology, Santa Barbara, CA, USA). V_H_/V_L_ sdAbs (10–20 µg) were injected at 30°C in a mobile phase consisting of calcium- and magnesium-free DPBS (GE Healthcare) at a flow rate of 0.4 mL/min. Weighted average molecular mass (*M*_MALS_) was calculated using a protein concentration determined using A_280_ from the PDA detector with extinction coefficients calculated from amino acid sequences. Data were processed using ASTRA 6.1 software (Wyatt).

### Surface Plasmon Resonance (SPR)

All monomeric human V_H_/V_L_ sdAbs were SEC-purified and buffer-exchanged into HBS-EP buffer (10 mM HEPES, 150 mM NaCl, 3 mM EDTA, 0.005% (v/v) surfactant P20, pH 7.4) prior to SPR analyses. All SPR analyses were performed on a Biacore™ 3000 instrument (GE Healthcare) at a temperature of 25°C. Briefly, proteins (CEACAM6, 370 resonance units (RUs); HSA, 1614 RUs; GITR, 796 RUs; MBP-intimin, 1536 RUs) were immobilized *via* amine coupling on research-grade CM5 sensor chips (GE Healthcare) in 10 mM acetate buffer, pH 4.0, according to the manufacturer’s instructions. V_H_/V_L_ sdAbs were injected at concentrations ranging from 1 nM to 10 µM, at flow rates of 20–50 µL/min and with contact time between 120 s and 300 s, then allowed to dissociate for 7–10 min. All surfaces were regenerated using 10 mM glycine, pH 1.5. Ethanolamine-blocked flow cells served as reference surfaces. The data were fitted to a 1:1 interaction model and binding affinities (*K*_D_s) and/or kinetic parameters were determined either by steady-state analysis or by multicycle kinetic analysis using BIAevaluation 4.1 software (GE Healthcare).

Surface plasmon resonance analyses of two V_L_ sdAbs (VL_SS_-2 and VL_SS_-5) against MBP-intimin were conducted as described above, except that 234 RUs of MBP-intimin were immobilized on a research-grade C1 sensor chip (GE Healthcare) *via* amine coupling in 10 mM acetate buffer, pH 4.5, according to the manufacturer’s instructions. These two V_L_ sdAbs were injected in HBS-EP buffer containing either 150 or 500 mM NaCl with an extended contact time (600 s).

### Protein Turbidity Assays

V_H_/V_L_ sdAb samples (0.2 mL) in 1.5 mL microcentrifuge tubes were heated to 85°C in a heating block for 10 min, then allowed to cool to room temperature for 30 min. Absorbance at 360 nm was measured pre- and post-heat treatment in a quartz NanoQuant Plate™ (Tecan, Männedorf, Switzerland) using an Infinite^®^ M200 PRO microplate reader (Tecan). V_H_/V_L_ sdAb concentrations were adjusted to 0.25 mg/mL in PBS prior to analysis.

### Thermal Shift Assays

V_H_/V_L_ sdAb samples (45 µL) in 96-well thin-wall optical plates (Bio-Rad) were mixed with 5 µL SYPRO^®^ Orange (diluted 1:100 from 5,000 × stock; Life Technologies, Carlsbad, CA, USA) and sealed with optical quality sealing tape (Bio-Rad). Using an iQ™ 5 real-time PCR system (Bio-Rad), a temperature ramp of 1°C/min was applied and thermal unfolding was monitored by measuring fluorescence at 0.5°C intervals as previously described (46, 47). The wavelengths for excitation and emission were 490 and 575 nm, respectively. Melting temperatures (*T*_m_s) were calculated as the temperature at which the maximum rate of change in fluorescent signal (d(RFU)/d*t*) was achieved. V_H_/V_L_ sdAb concentrations were adjusted to 1 mg/mL in PBS prior to analysis.

### Circular Dichroism

*T*_m_s were also determined by circular dichroism as previously described (39, 40). Ellipticity of V_H_/V_L_ sdAbs (100 µg/mL) was measured at wavelengths between 205 and 210 nm in 100 mM sodium phosphate buffer, pH 7.4. Ellipticity measurements were normalized to a percentage scale and then *T*_m_s were determined by plotting percent folded versus temperature and fitting the data to a Boltzmann distribution.

### Next-Generation DNA Sequencing

V_H_/V_L_ sdAb libraries were interrogated using an Illumina MiSeq instrument as described previously (44, 45, 48). Amplicons for NGS were prepared using FR1- and FR4-specific barcoded primers (sequences in Table S1 in Supplementary Material) using as template either phagemid DNA (~10 ng) or phage virions (~10^6^ particles).

### Statistical Analyses

Descriptive statistics (mean, median) were used to describe datasets as described in figure legends. No inferential statistical tests were used.

## Results

Antigen-specific human autonomous V_H_/V_L_ sdAbs do not exist in nature, and are most commonly isolated from synthetically randomized *in vitro* display libraries. These molecules are notoriously unstable and aggregation prone (4), which probably negatively impacts the selection of antigen-specific binders from synthetic V_H_/V_L_ sdAb libraries. In an effort to mitigate these factors, we attempted to identify ultra-stable V_H_/V_L_ sdAb scaffolds upon which we could construct highly diverse, stability-enhanced phage-displayed V_H_/V_L_ sdAb libraries designed to yield soluble and thermostable antigen-specific human sdAbs.

### Biophysical Stability Assessment of Wild-Type Human V_H_/V_L_ sdAb Scaffolds

The *T*_m_s and aggregation tendencies of 22 potential V_H_ sdAb scaffolds and 18 potential V_L_ sdAb scaffolds were determined by circular dichroism and SEC (Tables S2 and S3 in Supplementary Material). Five V_H_ and five V_L_ sdAb scaffolds as well as their disulfide-stabilized equivalents (Table 1) were selected as the most promising candidates for library construction based on: (i) primarily monomeric folding, (ii) high thermostability with reversible thermal unfolding, and (iii) reasonable expression yields (>1 mg/L) from overnight *E. coli* TG1 cultures. As reported previously (39, 40), incorporation of a disulfide linkage spanning IMGT positions 54–78 increased the *T*_m_ of each scaffold by ~5–20°C but also had unpredictable effects on expression yields.

### Tolerance of Human V_H_/V_L_ sdAb Scaffolds to CDR Modification

As a preliminary investigation into which of the 20 V_H_/V_L_ sdAb scaffolds might best tolerate library randomization, we grafted 12 sets of exogenous CDRs (Table 2) into each scaffold and assessed the resulting molecules’ expression level (Bradford assay), aggregation tendency (SEC-MALS), *T*_m_ (thermal shift assay), and ability to refold after thermal denaturation (turbidity assay). For V_L_ sdAb scaffolds, the 12 sets of exogenous CDRs were derived from non-antigen-specific human V_L_ sdAbs isolated from previously constructed libraries with expression yields ≥10 mg/L and for V_H_ sdAb scaffolds, the 12 sets of exogenous CDRs were selected from either human V_H_ sdAbs of unknown antigen specificity isolated from previously constructed libraries or camelid V_H_H sdAbs with expression yields ≥3 mg/L.

**Figure 1:**
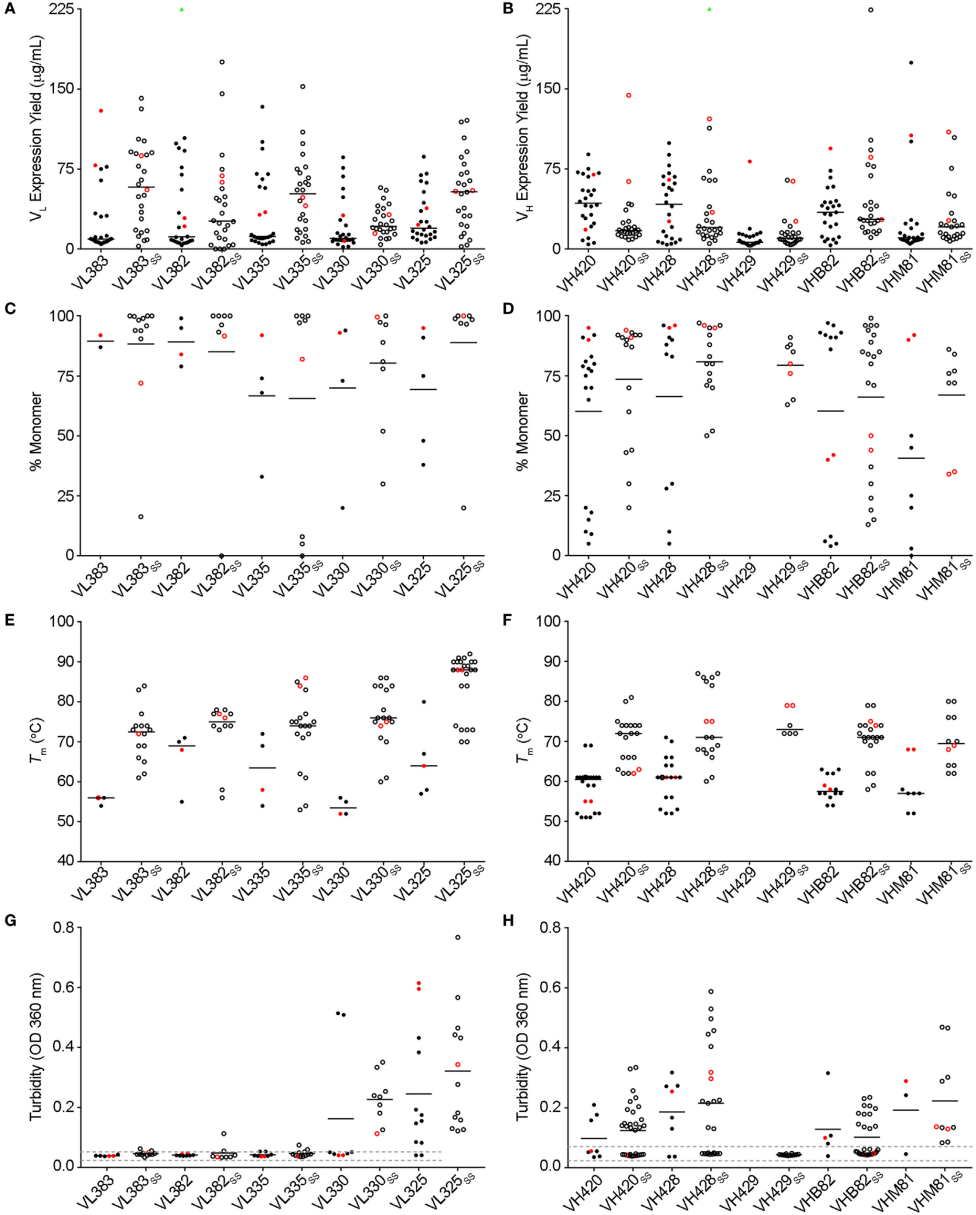
Biophysical stability assessment of complementarity-determining region (CDR)-shuffled human V_H_/V_L_ single-domain antibody (sdAb) variants. **(A,B)** Expression yields of CDR-shuffled V_L_ **(A)** and V_H_ **(B)** sdAbs from 5 mL overnight cultures, as determined by Bradford assay. Two outliers (VL382 + CDR set 9, expression yield 467.7 µg/mL and VH428_SS_ + CDR set 2, 356.4 µg/mL) are indicated by green triangles. **(C,D)** Aggregation tendencies of CDR-shuffled V_L_ **(C)** and V_H_ **(D)** sdAbs, as determined by SEC-MALS. **(E,F)** Melting temperatures (*T_m_*s) of CDR-shuffled V_L_ **(E)** and V_H_ **(F)** sdAbs, as determined by thermal shift assay. **(G,H)** Turbidity upon thermal denaturation of CDR-shuffled V_L_ **(G)** and V_H_ **(H)** sdAbs, as measured by spectrophotometry at 360 nm wavelength. Dashed lines represent the range of turbidity measurements for unheated V_H_/V_L_ sdAbs. The aggregation tendencies, *T_m_*s and turbidities of CDR-shuffled variants of VH429 were not characterized due to inadequate expression yields. Horizontal lines represent mean **(C,D,G,H)** or median **(A,B,E,F)** values and wild-type V_H_/V_L_ sdAbs with unmodified CDRs are shown in red. Open and solid circles represent V_H_/V_L_ sdAbs with, or without, a stabilizing exogenous disulfide linkage. The number of data points in each panel reflects whether the experiment was conducted in singlicate **(C,E)** or duplicate **(A,B,D,F–H)** and for panels **(C–H)**, whether the CDR-shuffled variant expressed sufficiently for analysis.

The V_L_ sdAb scaffolds yielding the best-expressing CDR-shuffled variants were VL383_SS_, VL325_SS_ and VL335_SS_ (Figure 1A). CDR-shuffled variants of VL383_SS_ and VL325_SS_ were primarily monomeric by SEC-MALS, while CDR-shuffled variants of VL335_SS_ showed some tendency to aggregate (Figure 1C). All three of these V_L_ sdAb scaffolds, especially VL325_SS_, yielded CDR-shuffled variants with high *T_m_*s (Figure 1E); however, many CDR-shuffled variants of VL325_SS_ showed significant turbidity upon heat denaturation (Figure 1G), reflecting their inability to refold as soluble monomers. The V_H_ sdAb scaffolds yielding the best-expressing CDR-shuffled variants were VH420, VH428, VHB82, VHB82_SS_, and VHM81_SS_ (Figure 1B). CDR-shuffled variants of all five of these V_H_ sdAb scaffolds were similarly monomeric (Figure 1D) but as expected, the two disulfide-stabilized sdAb scaffolds (VHB82_SS_ and VHM81_SS_) yielded CDR-shuffled variants with higher *T_m_*s (Figure 1F). Similar proportions of the CDR-shuffled variants of all five V_H_ sdAb scaffolds showed minor turbidity upon heat denaturation (Figure 1H). On the basis of these data, we selected one V_L_ sdAb scaffold (VL383_SS_) and two V_H_ sdAb scaffolds (VH428 and VHB82_SS_) for library construction, reflecting a balance of their tolerance to CDR modification as well as their differing CDR3 lengths and germline gene rearrangements.

### Design and Construction of Phage-Displayed Human V_H_/V_L_ sdAb Libraries

**Figure 2:**
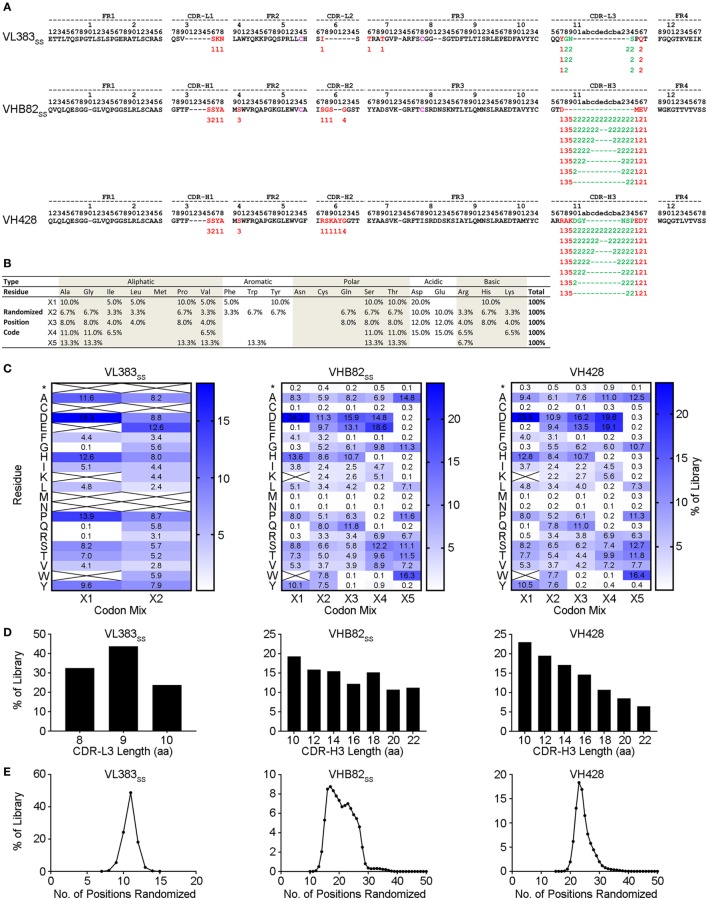
Design and construction of the VL383_SS_, VH428, and VHB82_SS_ synthetic human V_H_/V_L_ single-domain antibody (sdAb) libraries. **(A)** Design of the human V_H_/V_L_ sdAb libraries. The parental V_H_/V_L_ sdAb sequence is shown at top with IMGT numbering. Intradomain disulfide linkage-forming cysteine residues at positions 54 and 78 are shown in magenta. Randomization positions are highlighted in either red (no length polymorphism) or green (length polymorphism). Arabic numerals indicate the codon mixture (X1, X2, X3, X4, or X5) incorporated at each randomization position. **(B)** Expected amino acid frequencies at V_H_/V_L_ sdAb library randomization positions. **(C)** Observed amino acid frequencies at V_H_/V_L_ sdAb library randomization positions, measured from phagemid DNA isolated from *Escherichia coli* TG1 cells. Crossed-out cells indicate a frequency of <0.1%. **(D)** CDR3 length distributions of the V_H_/V_L_ sdAb libraries. **(E)** Degree of randomized sequence diversity (Levenshtein distance, or number of amino acid changes with respect to the parental scaffold) in the V_H_/V_L_ sdAb libraries. Analyses shown in **(C–E)** are representative of 7.7 × 10^4^ to 7.9 × 10^5^ sequences per library.

Three synthetic sdAb libraries (VL383_SS_, VH428, and VHB82_SS_) were constructed by limited *in vitro* randomization of the CDRs of these V_H_/V_L_ sdAb scaffolds and cloning of the resulting DNA into the pMED1 phagemid vector (Figures 2A,B). CDR3 length was varied in each library (Figures 2A,D; VL383_SS_ library: 8, 9, or 10 residues; VH428 and VHB82_SS_ libraries: 10, 12, 14, 16, 18, 20, or 22 residues) while CDR1 and CDR2 lengths were constant. The major technical advances of these over our previously described phage-displayed synthetic human V_H_/V_L_ sdAb libraries (12, 21, 31) were (i) trinucleotide mutagenesis allowed for incorporation of defined mixtures of amino acids at each randomization position, based on alignments of human and llama antibodies with additional bias toward solubility-promoting residues (Asp, Glu) and against undesirable residues (Asn, Cys, and Met) and stop codons (Figures 2B,C; Figure S2 in Supplementary Material) and (ii) near-complete randomization of all three CDRs of each V_H_/V_L_ sdAb scaffold resulted in much higher sequence diversity for the vast majority of library molecules (Figure 2E). All three V_H_/V_L_ sdAb libraries were transformed into *E. coli* TG1 cells, yielding final library sizes (1.0–2.3 × 10^10^ independent transformants) that, as expected, vastly under-sampled theoretical library diversity (VL383_SS_: 2.9 × 10^13^ unique sequences; VHB82_SS_: 2.3 × 10^33^ unique sequences; VH428: 2.3 × 10^35^ unique sequences). At this stage, prior to helper phage rescue, no major clonality biases or deviations from library design were observed (data not shown).

### Isolation and Characterization of Antigen-Specific Human V_H_/V_L_ sdAbs

**Figure 3:**
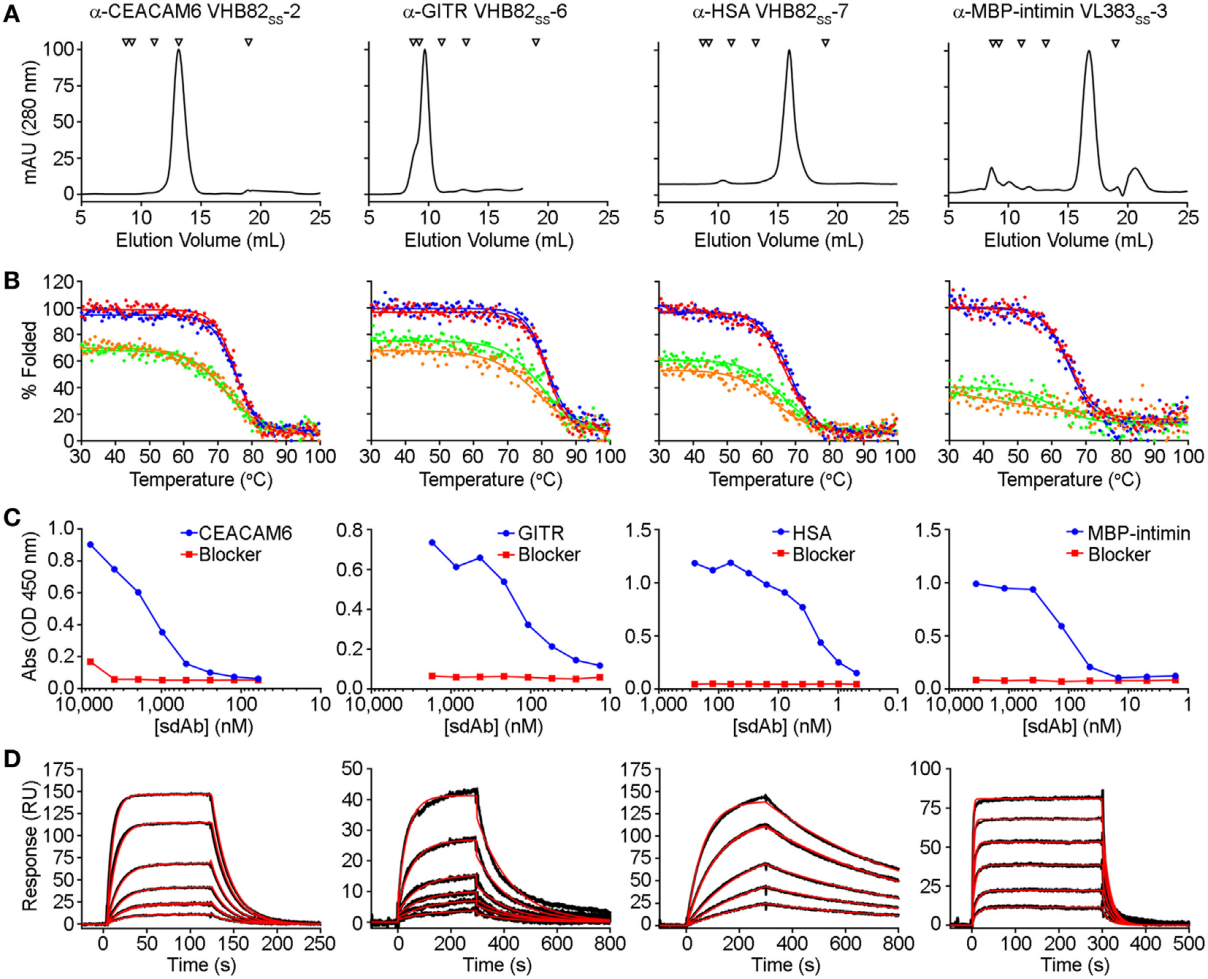
Properties of selected antigen-specific single-domain antibodies (sdAbs) isolated from the VL383_SS_, VH428, and VHB82_SS_ synthetic human V_H_/V_L_ sdAb libraries. The single highest affinity sdAb against each target is shown, except for MBP-intimin, where VL_SS_-3 is shown instead of VL_SS_-4 or VL_SS_-5 because of better fit to a 1:1 binding model. **(A)** Size exclusion chromatography (SEC) profiles of representative antigen-specific human V_H_/V_L_ sdAbs. Arrows show molecular mass standards, from left to right: thyroglobulin (670 kDa), gamma globulin (158 kDa), ovalbumin (44 kDa), myoglobin (17 kDa), and vitamin B12 (1.35 kDa). **(B)** Thermal unfolding of representative antigen-specific V_H_/V_L_ sdAbs as determined by circular dichroism. Replicate unfolding curves are shown in red and blue; the sdAbs were then cooled to room temperature and remelted (shown in orange and green). **(C)** Titration ELISA of representative antigen-specific V_H_/V_L_ sdAbs. Horseradish peroxidase mouse anti-c-Myc secondary antibody (clone 9E10, diluted 1:3,000) was used for detection. **(D)** Binding of antigen-specific V_H_/V_L_ sdAbs to cognate antigen by SPR. Each antigen was immobilized on a CM5 sensor chip using amine coupling, then the indicated V_H_ or V_L_ sdAb was flowed over the surface at concentrations ranging from 25 to 1,000 nM (α-CEACAM6 VHB82_SS_-2), 25 to 500 nM (α-GITR VHB82_SS_-6), 2.5 to 50 nM (α-HSA VHB82_SS_-7), or 62.5 to 2,500 nM (α-MBP-intimin VL383_SS_-3). Black lines show data and red lines show fits.

**Figure 4:**
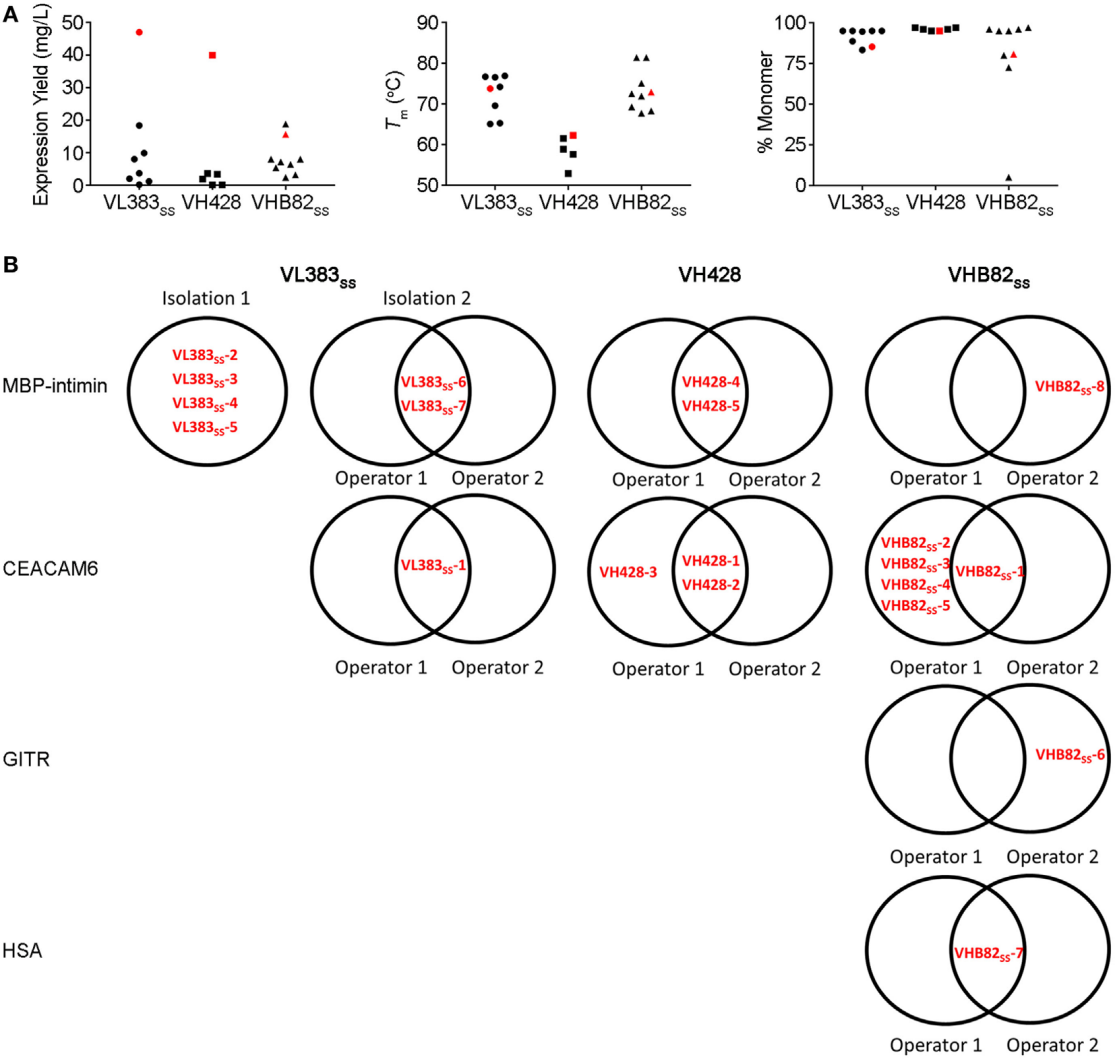
Properties of all antigen-specific single-domain antibodies (sdAbs) isolated from the VL383_SS_, VH428 and VHB82_SS_ synthetic human V_H_/V_L_ sdAb libraries. **(A)** Expression yields (as measured by spectrophotometry at 280 nm), *T_m_*s (as determined by circular dichroism), and aggregation tendencies (as determined by size exclusion chromatography) of antigen-specific V_H_/V_L_ sdAbs isolated from the libraries in comparison to the parental scaffold from which they were derived (shown in red). **(B)** Interoperator reproducibility in isolating individual V_H_/V_L_ sdAb sequences.

All three V_H_/V_L_ sdAb libraries were rescued with M13K07 helper phage and panned for four or five rounds against five model antigens (CEACAM6, CTLA4, GITR, HSA, and MBP-intimin). All library pannings were performed in duplicate by two independent operators at the same time and using the same materials, except for panning against MBP-intimin, in which panning was done in triplicate. High attrition rates of V_H_/V_L_ sdAbs showing false positive binding either as sdAb-phage or as soluble sdAb proteins (VL383_SS_: 53% attrition; VH428: 69% attrition; VHB82ss: 71% attrition; Table 3) resulted in a final yield of 20 unique antigen-specific V_H_/V_L_ sdAbs against four targets. Of the 15 library-target screens we conducted, eight (53%) yielded at least one antigen-specific V_H_/V_L_ sdAb (four screens yielded one sdAb, two screens yielded two sdAbs, one screen yielded three sdAbs, and two screens yielded five sdAbs). Most of the recovered sdAbs were monomeric (Figure 3A; Table 4; Figure S3 in Supplementary Material), thermostable (Figure 3B; Table 4), and had affinities for antigen ranging from 5 nM to ~12 μM (Figures 3C,D; Table 4). One notable exception was the α-GITR V_H_ sdAb VHB82_SS_-6, whose elution volume by SEC suggested it existed in solution as a strict dimer (Figure 3A). Stable homodimerization has previously been reported for several human V_H_ sdAbs and was critical for antigen binding (49–52); this phenomenon is distinct from the general tendency of some V_H_ domains to forms soluble aggregates. Antigen-specific V_H_/V_L_ sdAbs isolated from all three libraries had aggregation tendencies and thermostabilities reflective of the parental V_H_/V_L_ sdAb scaffold from which they were derived, but generally poorer expression yields (Figure 4A). Reproducibility in the selection of specific sdAb sequences from the libraries was modest, with only 45% (9/20) of antigen-specific V_H_/V_L_ sdAbs recovered by both operators (Figure 4B) and no apparent connection between antigen-binding affinity and consistency of isolation.

**Table 3:** Attrition rates of human V_H_/V_L_ single-domain antibodies (sdAbs) through isolation and characterization.

Library	Target	No. of binding sdAbs		
		Phage ELISA	Soluble ELISA	SPR
VL383_SS_	CEACAM6	1	1	1
	CTLA4	4	0	
	GITR	0		
	HSA	2	0	
	MBP-intimin^a^	3, 5	2, 4	2, 4
		15 (100%)	7 (47%)	7 (47%)
VH428	CEACAM6	3	3	3
	CTLA4	3	0	
	GITR	4	0	
	HSA	2	0	
	MBP-intimin	4	2	2
		16 (100%)	5 (31%)	5 (31%)
VHB82_SS_	CEACAM6	5	5	5
	CTLA4	9	0	
	GITR	5	1	1
	HSA	6	3	1
	MBP-intimin	3	2	1
		28 (100%)	11 (39%)	8 (29%)

Total		59 (100%)	23 (39%)	20 (34%)

*^a^Double entries reflect two independent isolation attempts*.

**Table 4:** Properties of antigen-specific single-domain antibodies (sdAbs) isolated from the phage-displayed synthetic human V_H_/V_L_ libraries.

Target	V_H_/V_L_ sdAb	Expression yield (mg/L)	Monomer (%)	*T*_m_ (°C)	*K*_D_ (nM)
CEACAM6	VL383_SS_-1	18.4	>95	76.9	454^a^
	VH428-1	3.4	>95	57.6	2,180^a^
	VH428-2	3.6	>95	61.5	1,230^b^
	VH428-3	1.9	>95	52.9	290^b^
	VHB82_SS_-1	6.4	>95	81.4	638^b^
	VHB82_SS_-2	18.8	>95	75.1	245^b^
	VHB82_SS_-3	5.4	>95	72.5	2,730^a^
	VHB82_SS_-4	3.2	80.0	68.3	1,070^a^
	VHB82_SS_-5	2.4	>95	71.9	2,200^a^
GITR	VHB82_SS_-6	7.2	0^e^	81.4	196^b^
HSA	VHB82_SS_-7	8.0	>95	67.7	5^b^
MBP-intimin	VL383_SS_-2	2.0	>95	69.6	905^b,c^
	VL383_SS_-3	0.2	83.4	65.1	395^b^
	VL383_SS_-4	1.2	88.7	65.3	227^b^
	VL383_SS_-5	3.7	94.6	74.2	252^b,c^
	VL383_SS_-6	9.9	>95	76.6	7,470^a^
	VL383_SS_-7	8.0	>95	76.7	11,500^a,d^
	VH428-4	0.1	>95	58.9	2,590^b^
	VH428-5	0.1	>95	n.d.	4,430^a^
	VHB82_SS_-8	8.0	72.5	69.3	5,230^a^

*^a^Determined using steady-state analysis at 25°C*.

*^b^Determined using multicycle kinetic analysis and fitting to a 1:1 binding model at 25°C*.

*^c^Antigen was immobilized on a C1 sensor chip, evidence of complex binding*.

*^d^K_D_ is an estimate only due to lack of curvature in the steady-state plot*.

*^e^Size exclusion chromatography elution volume suggests VHB82_SS_-6 is a strict dimer*.

*n.d., not determined*.

### Evaluation of Stability-Sequence Diversity Tradeoffs in Human V_H_/V_L_ sdAb Libraries

**Figure 5:**
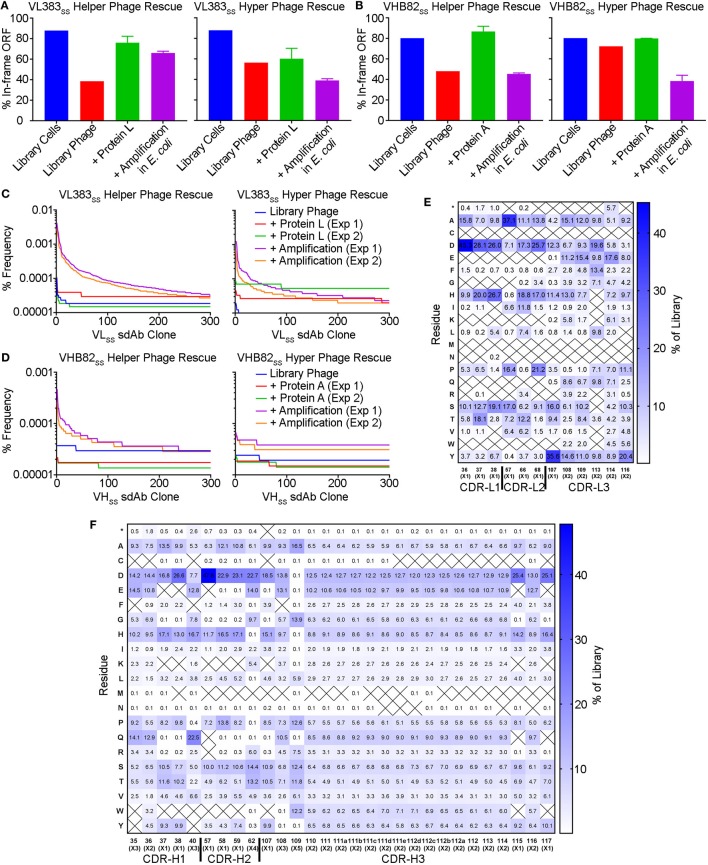
Impact of stability selection on human V_H_/V_L_ single-domain antibody (sdAb) library sequence diversity. After transformation of *Escherichia coli* TG1 cells with phagemid DNA, phage were rescued by superinfection of overnight cultures with M13K07 helper phage or M13K07ΔpIII hyper phage. The purified sdAb-displaying phage were bound and eluted from protein A (VHB82_SS_ library) or protein L (VL383_SS_ library), then amplified by reinfection of *E. coli* TG1 cells. Three rounds of panning were performed, and V_H_/V_L_ sdAb sequences were interrogated by NGS at the stage of rescued phage, phage eluted after a single round of protein A/L selection, and phage amplified after the final round of panning. **(A,B)** Proportion of functional sdAb sequences (in-frame ORF, no stop codons) observed in V_L_ **(A)** and V_H_ **(B)** library phage and panning outputs. **(C,D)** Clonality of V_L_ **(C)** and V_H_ **(D)** library phage and panning outputs. **(E,F)** Complementarity-determining region (CDR) amino acid composition of enriched V_L_ **(E)** and V_H_ **(F)** sdAb clones after three rounds of stability selection. Crossed-out cells indicate a frequency of <0.1%. Analyses in **(A–F)** are representative of 7.7 × 10^4^ to 7.9 × 10^5^ sequences per sample.

To better understand potential constraints on the CDR sequences of monomeric and stable human V_H_/V_L_ sdAbs, we examined the effects of stability selection (three rounds of protein A or L selection followed by amplification of the eluted phage overnight in *E. coli* TG1 cells; Figure 5) on randomized sequence diversity of two V_H_/V_L_ sdAb libraries (VL383_SS_ and VHB82_SS_). We performed this experiment in duplicate using either phage particles displaying monovalent sdAb (rescued with M13K07 helper phage; simultaneous expression of pIII from helper phage and pIII-sdAb from phagemid) or phage particles displaying multivalent sdAb (rescued with M13K07ΔpIII hyper phage; only pIII-sdAb expressed from phagemid). Out-of-frame and/or stop-codon-encoding V_H_/V_L_ sdAb clones were rare in both libraries (“Library Cells”), but rose substantially in frequency upon phage rescue (“Library Phage”) and upon amplification in *E. coli* (“Amplification in *E. coli*”), suggesting a growth advantage for non-sdAb expressing cells over sdAb-expressing ones (Figures 5A,B); this phenomenon was mitigated somewhat using hyper phage rescue. After three rounds of protein A/L selection and overnight amplification in *E. coli* with helper phage rescue, there were clear clonal biases observed in the resulting populations of sdAb-phage (Figures 5C,D). Using an arbitrary frequency cutoff (0.00006% for VL383_SS_ and 0.00004% for VHB82_SS_; sdAbs at frequencies greater than these cutoffs were not present in any other dataset) to identify unique sdAb sequences enriched by stability selection, we found that different sdAb clones were selected in the two replicate pannings, although both sets of sdAbs were heavily biased toward the parental V_H_/V_L_ sdAb scaffold’s CDR3 length (9 residues for the VL383_SS_ library and 10 residues for the VHB82_SS_ library, which is the shortest CDR3 length possible in the library design and the nearest to the VHB82_SS_ scaffold’s CDR3 length of 6 residues; Figure S4 in Supplementary Material). However, stability selection reproducibly yielded V_H_/V_L_ sdAbs with biased CDR amino acid composition (Figures 5E,F; Figures S5 and S6 in Supplementary Material). In order of magnitude, stability biases in the VL383_SS_ library favored Asp, His, Pro, Ser and Thr and disfavored aromatic (Phe, Trp, Tyr) and hydrophobic (Ile, Leu, Val) residues. Similarly, stability biases in the VHB82_SS_ library favored Asp, His, Pro, and Gln and disfavored aromatic (Phe, Trp, Tyr) and hydrophobic (Ile, Leu, Val) residues. These biases were especially acute at the C-terminus of CDR1 and N-terminus of CDR2, both of which flank FR2. Identical molecular signatures were observed to a lesser degree in the pools of all sdAb-phage (irrespective of frequency or mono- vs. multivalent display format) subjected to three rounds of stability selection (Figures S7 and S8 in Supplementary Material) as well as in the pools of sdAb-phage after phage rescue and eluted from protein A/L (data not shown), suggesting a common ontogeny.

## Discussion

The starting point for this investigation was the observation that several of our previously described synthetic human V_H_/V_L_ sdAb libraries performed unpredictably (12, 21, 31), yielding monomeric antigen-specific binders against some targets but not others. Thus, we tested the hypothesis that these issues might be solved by: (i) building libraries on ultrastable V_H_/V_L_ sdAb scaffolds that maintain their biophysical properties upon CDR modification and (ii) randomizing the CDRs of the V_H_/V_L_ sdAb libraries more extensively using trinucleotide mutagenesis. A few general lessons became apparent during the course of these experiments. First, although V_H_/V_L_ sdAb scaffolds clearly vary in their tolerance to CDR modification, no “perfect” scaffold exists (i.e., some CDR-shuffled variants of every scaffold showed poor biophysical properties). Second, the trinucleotide mutagenesis approach used here was dramatically more effective at incorporating randomized sequence diversity in V_H_/V_L_ sdAb CDRs compared to the Kunkel mutagenesis (21, 31) and splicing by overlap extension PCR mutagenesis approaches using degenerate oligonucleotides (12) that we have used previously. Third, as we showed previously (31), incorporating a stabilizing exogenous disulfide linkage spanning IMGT positions 54–78 into V_H_/V_L_ sdAb libraries is clearly compatible with selection of antigen-specific binders, as 15/20 of the binders shown here bear this linkage. Finally, it is clearly possible, at least under some circumstance, to select monomeric, high-affinity binders from synthetic human V_H_/V_L_ sdAbs libraries, as evidenced by the α-HSA VH_SS_-7 sdAb (*K*_D_: 5 nM) reported here.

However, the more general issues afflicting synthetic human V_H_/V_L_ sdAb libraries (high attrition rates due to false positive binding, possibly caused by sdAb aggregation; inconsistency of isolation; variable expression yield and aggregation of isolated sdAbs) were not completely solved by using more stable V_H_/V_L_ scaffolds or building more diverse and non-degenerate libraries. The additional randomized sequence diversity we achieved by the use of trinucleotide mutagenesis is almost certainly negligible when compared to the theoretical size of the libraries, and massive undersampling may account at least in part for inconsistent isolation of sdAbs. Other potential solutions to the problem of undersampling include increasing throughput (53), use of V_H_/V_L_ sdAb-transgenic mice (54, 55) and use of *in vitro* VDJ recombination systems (56). Nonetheless, modest inter-operator reproducibility in isolating the same binders clearly demonstrates that antigen-specific human V_H_/V_L_ sdAbs are not always isolated even when they were present in the library. We currently have no understanding of the factors influencing the number of V_H_/V_L_ sdAb binders isolated, their affinities or biophysical properties, nor if these depend on the library, the target antigen quality or composition, or the panning methodology. One possibility, for example, is that the numbers of input library sdAb-phage used here, the target antigen surface size and density, and/or the numbers of eluted phage amplified from each round of selection were inadequate to consistently recover very rare sdAb specificities that may be present in the libraries. These factors could be investigated empirically in future studies.

It is virtually certain that some of the challenges of human synthetic V_H_/V_L_ sdAbs relate to fundamental tradeoffs between stability and sequence diversity. There is no necessary reason why rare autonomous rearranged human V_H_/V_L_ sdAbs should be compatible with any CDR sequence; rather, it should be expected that these molecules rely chiefly on particular CDR sequences for their solubility and stability, given that human V_H_ and V_L_ domains have evolved to be paired with one another, occluding a hydrophobic surface between the two domains. We expect that such challenges are much less of a problem for protein domains that naturally exist as soluble monomers such as camelid and shark sdAbs and non-antibody scaffolds (e.g., FN3 and SH3 domains). Some camelid V_H_Hs rely on CDR residues for solubility as well (57), albeit likely to a lesser extent due to the presence of solubility-enhancing FR2 residues. One obvious explanation for the previously described bias toward negatively charged residues resulting in acidic overall pIs of V_H_/V_L_ sdAbs (4, 12), especially in CDR1 and CDR2 (10, 11), is that this may enhance solubility and aggregation resistance. The role of charge and negative charge in particular in solubilizing V_H_/V_L_ sdAbs with hydrophobic CDRs has been previously demonstrated (58, 59), and appears to be highly position and scaffold dependent (4). Bias in favor of His residues is less easy to explain, although its imidazole side chain may also have stabilizing and solubilizing effects near physiological pH (60). Notably, “camelized” human V_H_ sdAbs and human V_H_/V_L_ sdAbs selected *in vitro* to bear solubility-promoting FR substitutions (9, 61), may not be subject to the same stability-sequence diversity tradeoffs. We caution that although the growth advantages conferred by V_H_/V_L_ sdAbs with the most stable and soluble CDR sequences were most apparent using helper phage rescue of phagemid libraries, forcing pIII-sdAb expression using hyper phage rescue or by using phage (not phagemid) or yeast display systems would not be expected to circumvent the issue of bias; instead, it might be expected to result in immediate loss of a large population of V_H_/V_L_ sdAbs with poorly stable and soluble CDR sequences.

In conclusion, we have described three novel synthetic human V_H_/V_L_ sdAb libraries as well as antigen-specific binders against a variety of target antigens selected from these libraries. Future work will seek to better understand the constraints imposed on human V_H_/V_L_ sdAbs by stability-sequence diversity tradeoffs, and whether it is possible to circumvent them by *in vitro* engineering of human autonomous immunoglobulin variable domain folds. One possibility would be to identify and fix a minimal set of stability-enhancing CDR residues in human V_H_/V_L_ sdAb libraries, which might allow for more effective randomization of the remaining CDR positions. However, doing so without significant divergence from human germline IGHV sequences, increasing the risk of immunogenicity, may be very challenging.

## Author Contributions

DK, HK, and JT isolated and characterized human V_H_/V_L_ sdAb scaffolds and conducted CDR-shuffling experiments and designed the libraries. JS conducted SEC-MALS experiments. ML, HK, and KH isolated and characterized antigen-specific sdAbs. ML and KH conceived and carried out stability selections and next-generation DNA sequencing analyses. QY, GH, and CRM designed, carried out, and analyzed surface plasmon resonance experiments. KH and GH made the figures and KH wrote the manuscript. All authors read and approved the final manuscript.

## Conflict of Interest Statement

JT and DK are inventors of US patents 8293233B2 and 9371371B2 as well as other patents and patent applications governing human V_H_/V_L_ sdAb scaffolds.

## References

[1] Ward ES Gussow D Griffiths AD Jones PT Winter G. Binding activities of a repertoire of single immunoglobulin variable domains secreted from *Escherichia coli*. Nature (1989) 341:544–6. 10.1038/341544a0 2677748

[2] Hamers-Casterman C Atarhouch T Muyldermans S Robinson G Hamers C Songa EB Naturally occurring antibodies devoid of light chains. Nature (1993) 363:446–8. 10.1038/363446a0 8502296

[3] Greenberg AS Avila D Hughes M Hughes A McKinney EC Flajnik MF. A new antigen receptor gene family that undergoes rearrangement and extensive somatic diversification in sharks. Nature (1995) 374:168–73. 10.1038/374168a0 7877689

[4] Kim DY Hussack G Kandalaft H Tanha J. Mutational approaches to improve the biophysical properties of human single-domain antibodies. Biochim Biophys Acta (2014) 1844:1983–2001. 10.1016/j.bbapap.2014.07.008 25065345

[5] Tanha J Nguyen TD Ng A Ryan S Ni F MacKenzie R Improving solubility and refolding efficiency of human V_H_s by a novel mutational approach. Protein Eng Des Sel (2006) 19:503–9. 10.1093/protein/gzl037 16971398

[6] Tanha J Xu P Chen Z Ni F Kaplan H Narang SA Optimal design features of camelized human single-domain antibody libraries. J Biol Chem (2001) 276:24774–80. 10.1074/jbc.M100770200 11335716

[7] Davies J Riechmann L. Single antibody domains as small recognition units: design and *in vitro* antigen selection of camelized, human V_H_ domains with improved protein stability. Protein Eng (1996) 9:531–7. 10.1093/protein/9.6.531 8862554

[8] Davies J Riechmann L. Antibody V_H_ domains as small recognition units. Biotechnology (N Y) (1995) 13:475–9. 10.1038/nbt0595-475 9634788

[9] Ma X Barthelemy PA Rouge L Wiesmann C Sidhu SS. Design of synthetic autonomous V_H_ domain libraries and structural analysis of a V_H_ domain bound to vascular endothelial growth factor. J Mol Biol (2013) 425:2247–59. 10.1016/j.jmb.2013.03.020 23507309

[10] Dudgeon K Famm K Christ D. Sequence determinants of protein aggregation in human V_H_ domains. Protein Eng Des Sel (2009) 22:217–20. 10.1093/protein/gzn059 18957405

[11] Dudgeon K Rouet R Kokmeijer I Schofield P Stolp J Langley D General strategy for the generation of human antibody variable domains with increased aggregation resistance. Proc Natl Acad Sci U S A (2012) 109:10879–84. 10.1073/pnas.1202866109 22745168 PMC3390889

[12] Arbabi-Ghahroudi M To R Gaudette N Hirama T Ding W MacKenzie R Aggregation-resistant V_H_s selected by *in vitro* evolution tend to have disulfide-bonded loops and acidic isoelectric points. Protein Eng Des Sel (2009) 22:59–66. 10.1093/protein/gzn071 19033278

[13] Christ D Famm K Winter G. Repertoires of aggregation-resistant human antibody domains. Protein Eng Des Sel (2007) 20:413–6. 10.1093/protein/gzm037 17720749

[14] Christ D Famm K Winter G Tapping diversity lost in transformations – *in vitro* amplification of ligation reactions. Nucleic Acids Res (2006) 34:e108 10.1093/nar/gkl605 16945952 PMC1636367

[15] De Bernardis F Liu H O’Mahony R La Valle R Bartollino S Sandini S Human domain antibodies against virulence traits of *Candida albicans* inhibit fungus adherence to vaginal epithelium and protect against experimental vaginal candidiasis. J Infect Dis (2007) 195:149–57. 10.1086/509891 17152019

[16] Pinelli DF Wagener ME Liu D Yamniuk A Tamura J Grant S An anti-CD154 domain antibody prolongs graft survival and induces Foxp3(+) iTreg in the absence and presence of CTLA-4 Ig. Am J Transplant (2013) 13:3021–30. 10.1111/ajt.12417 24007441 PMC4287239

[17] Suchard SJ Davis PM Kansal S Stetsko DK Brosius R Tamura J A monovalent anti-human CD28 domain antibody antagonist: preclinical efficacy and safety. J Immunol (2013) 191:4599–610. 10.4049/jimmunol.1300470 24081989

[18] Yamniuk AP Suri A Krystek SR Tamura J Ramamurthy V Kuhn R Functional antagonism of human CD40 achieved by targeting a unique species-specific epitope. J Mol Biol (2016) 428:2860–79. 10.1016/j.jmb.2016.05.014 27216500

[19] Walker A Chung CW Neu M Burman M Batuwangala T Jones G Novel interaction mechanism of a domain antibody-based inhibitor of human vascular endothelial growth factor with greater potency than ranibizumab and bevacizumab and improved capacity over aflibercept. J Biol Chem (2016) 291:5500–11. 10.1074/jbc.M115.691162 26728464 PMC4786692

[20] Xie JH Yamniuk AP Borowski V Kuhn R Susulic V Rex-Rabe S Engineering of a novel anti-CD40L domain antibody for treatment of autoimmune diseases. J Immunol (2014) 192:4083–92. 10.4049/jimmunol.1303239 24670803

[21] Hussack G Keklikian A Alsughayyir J Hanifi-Moghaddam P Arbabi-Ghahroudi M van Faassen H A V_L_ single-domain antibody library shows a high-propensity to yield non-aggregating binders. Protein Eng Des Sel (2012) 25:313–8. 10.1093/protein/gzs014 22490957

[22] Zhang H Yun S Batuwangala TD Steward M Holmes SD Pan L A dual-targeting antibody against EGFR-VEGF for lung and head and neck cancer treatment. Int J Cancer (2012) 131:956–69. 10.1002/ijc.26427 21918971

[23] Li N Fu H Hewitt SM Dimitrov DS Ho M. Therapeutically targeting glypican-2 via single-domain antibody-based chimeric antigen receptors and immunotoxins in neuroblastoma. Proc Natl Acad Sci U S A (2017) 114:E6623–31. 10.1073/pnas.1706055114 28739923 PMC5559039

[24] Feng M Gao W Wang R Chen W Man YG Figg WD Therapeutically targeting glypican-3 via a conformation-specific single-domain antibody in hepatocellular carcinoma. Proc Natl Acad Sci U S A (2013) 110:E1083–91. 10.1073/pnas.1217868110 23471984 PMC3607002

[25] Jespers L Schon O Famm K Winter G. Aggregation-resistant domain antibodies selected on phage by heat denaturation. Nat Biotechnol (2004) 22:1161–5. 10.1038/nbt1000 15300256

[26] Walker A Dunlevy G Rycroft D Topley P Holt LJ Herbert T Anti-serum albumin domain antibodies in the development of highly potent, efficacious and long-acting interferon. Protein Eng Des Sel (2010) 23:271–8. 10.1093/protein/gzp091 20093262

[27] Holt LJ Basran A Jones K Chorlton J Jespers LS Brewis ND Anti-serum albumin domain antibodies for extending the half-lives of short lived drugs. Protein Eng Des Sel (2008) 21:283–8. 10.1093/protein/gzm067 18387938

[28] Rouet R Dudgeon K Christie M Langley D Christ D. Fully human V_H_ single domains that rival the stability and cleft recognition of camelid antibodies. J Biol Chem (2015) 290:11905–17. 10.1074/jbc.M114.614842 25737448 PMC4424330

[29] Mandrup OA Friis NA Lykkemark S Just J Kristensen P. A novel heavy domain antibody library with functionally optimized complementarity determining regions. PLoS One (2013) 8:e76834. 10.1371/journal.pone.0076834 24116173 PMC3792991

[30] Jespers L Schon O James LC Veprintsev D Winter G Crystal structure of HEL4, a soluble, refoldable human V_H_ single domain with a germ-line scaffold. J Mol Biol (2004) 337:893–903. 10.1016/j.jmb.2004.02.013 15033359

[31] Henry KA Kandalaft H Lowden MJ Rossotti MA van Faassen H Hussack G A disulfide-stabilized human V_L_ single-domain antibody library is a source of soluble and highly thermostable binders. Mol Immunol (2017) 90:190–6. 10.1016/j.molimm.2017.07.006 28820969

[32] Yu GW Vaysburd M Allen MD Settanni G Fersht AR. Structure of human MDM4 N-terminal domain bound to a single-domain antibody. J Mol Biol (2009) 385:1578–89. 10.1016/j.jmb.2008.11.043 19084022

[33] Tang Z Feng M Gao W Phung Y Chen W Chaudhary A A human single-domain antibody elicits potent antitumor activity by targeting an epitope in mesothelin close to the cancer cell surface. Mol Cancer Ther (2013) 12:416–26. 10.1158/1535-7163.MCT-12-0731 23371858 PMC3624043

[34] Gay RD Clarke AW Elgundi Z Domagala T Simpson RJ Le NB Anti-TNF-α domain antibody construct CEP-37247: full antibody functionality at half the size. MAbs (2010) 2:625–38. 10.4161/mabs.2.6.13493 20930515 PMC3011217

[35] Bertok S Wilson MR Morley PJ de Wildt R Bayliffe A Takata M. Selective inhibition of intra-alveolar p55 TNF receptor attenuates ventilator-induced lung injury. Thorax (2012) 67:244–51. 10.1136/thoraxjnl-2011-200590 22156959 PMC3282043

[36] Holland MC Wurthner JU Morley PJ Birchler MA Lambert J Albayaty M Autoantibodies to variable heavy (V_H_) chain Ig sequences in humans impact the safety and clinical pharmacology of a V_H_ domain antibody antagonist of TNF-α receptor 1. J Clin Immunol (2013) 33:1192–203. 10.1007/s10875-013-9915-0 23832582

[37] Cordy JC Morley PJ Wright TJ Birchler MA Lewis AP Emmins R Specificity of human anti-variable heavy (V_H_) chain autoantibodies and impact on the design and clinical testing of a V_H_ domain antibody antagonist of tumour necrosis factor-α receptor 1. Clin Exp Immunol (2015) 182:139–48. 10.1111/cei.12680 26178412 PMC4608503

[38] To R Hirama T Arbabi-Ghahroudi M MacKenzie R Wang P Xu P Isolation of monomeric human V_H_s by a phage selection. J Biol Chem (2005) 280:41395–403. 10.1074/jbc.M509900200 16221664

[39] Kim DY To R Kandalaft H Ding W van Faassen H Luo Y Antibody light chain variable domains and their biophysically improved versions for human immunotherapy. MAbs (2014) 6:219–35. 10.4161/mabs.26844 24423624 PMC3929445

[40] Kim DY Kandalaft H Ding W Ryan S van Faassen H Hirama T Disulfide linkage engineering for improving biophysical properties of human V_H_ domains. Protein Eng Des Sel (2012) 25:581–9. 10.1093/protein/gzs055 22942392

[41] Virnekas B Ge L Pluckthun A Schneider KC Wellnhofer G Moroney SE. Trinucleotide phosphoramidites: ideal reagents for the synthesis of mixed oligonucleotides for random mutagenesis. Nucleic Acids Res (1994) 22:5600–7. 10.1093/nar/22.25.5600 7838712 PMC310122

[42] Baral TN MacKenzie R Arbabi Ghahroudi M. Single-domain antibodies and their utility. Curr Protoc Immunol (2013) 103:Unit 2.17. 10.1002/0471142735.im0217s103 24510545

[43] Dorion-Thibaudeau J St-Laurent G Raymond C De Crescenzo G Durocher Y Biotinylation of the Fcγ receptor ectodomains by mammalian cell co-transfection: application to the development of a surface plasmon resonance-based assay. J Mol Recognit (2016) 29:60–9. 10.1002/jmr.2495 26762306

[44] Henry KA Hussack G Collins C Zwaagstra JC Tanha J MacKenzie CR Isolation of TGF-β-neutralizing single-domain antibodies of predetermined epitope specificity using next-generation DNA sequencing. Protein Eng Des Sel (2016) 29:439–43. 10.1093/protein/gzw043 27613412

[45] Henry KA Tanha J Hussack G. Identification of cross-reactive single-domain antibodies against serum albumin using next-generation DNA sequencing. Protein Eng Des Sel (2015) 28:379–83. 10.1093/protein/gzv039 26319004

[46] Lo MC Aulabaugh A Jin G Cowling R Bard J Malamas M Evaluation of fluorescence-based thermal shift assays for hit identification in drug discovery. Anal Biochem (2004) 332:153–9. 10.1016/j.ab.2004.04.031 15301960

[47] Ericsson UB Hallberg BM Detitta GT Dekker N Nordlund P. Thermofluor-based high-throughput stability optimization of proteins for structural studies. Anal Biochem (2006) 357:289–98. 10.1016/j.ab.2006.07.027 16962548

[48] Henry KA Sulea T van Faassen H Hussack G Purisima EO MacKenzie CR A rational engineering strategy for designing protein A-binding camelid single-domain antibodies. PLoS One (2016) 11:e0163113. 10.1371/journal.pone.0163113 27631624 PMC5025174

[49] Baral TN Chao SY Li S Tanha J Arbabi-Ghahroudi M Zhang J Crystal structure of a human single domain antibody dimer formed through V_H_-V_H_ non-covalent interactions. PLoS One (2012) 7:e30149 10.1371/journal.pone.0030149 22253912 PMC3257273

[50] Sepulveda J Jin H Sblattero D Bradbury A Burrone OR. Binders based on dimerised immunoglobulin V_H_ domains. J Mol Biol (2003) 333:355–65. 10.1016/j.jmb.2003.08.033 14529622

[51] Jin H Sepulveda J Burrone OR Selection and characterisation of binders based on homodimerisation of immunoglobulin V_H_ domains. FEBS Lett (2003) 554:323–9. 10.1016/S0014-5793(03)01182-7 14623088

[52] Jin H Sepulveda J Burrone OR. Specific recognition of a dsDNA sequence motif by an immunoglobulin V_H_ homodimer. Protein Sci (2004) 13:3222–9. 10.1110/ps.04921704 15557264 PMC2287315

[53] Wu Y Batyuk A Honegger A Brandl F Mittl PRE Plückthun A. Rigidly connected multispecific artificial binders with adjustable geometries. Sci Rep (2017) 7:11217. 10.1038/s41598-017-11472-x 28894181 PMC5593856

[54] Drabek D Janssens R de Boer E Rademaker R Kloess J Skehel J Expression cloning and production of human heavy-chain-only antibodies from murine transgenic plasma cells. Front Immunol (2016) 7:619. 10.3389/fimmu.2016.00619 28066429 PMC5165034

[55] Brüggemann M Zou X, Inventors; Crescendo Biologics Ltd., Assignee. Mouse λ Light Chain Locus. United States patent US 9439405 B2 (2016).

[56] Gallo M Kang JS Pigott CR, Inventors; Innovative Targeting Solutions, Inc., Assignee. Sequence Diversity Generation in Immunoglobulins. United States patent US 8012714 B2 (2011).

[57] Conrath K Vincke C Stijlemans B Schymkowitz J Decanniere K Wyns L Antigen binding and solubility effects upon the veneering of a camel V_H_H in framework-2 to mimic a V_H_. J Mol Biol (2005) 350:112–25. 10.1016/j.jmb.2005.04.050 15913651

[58] Perchiacca JM Ladiwala AR Bhattacharya M Tessier PM. Aggregation-resistant domain antibodies engineered with charged mutations near the edges of the complementarity-determining regions. Protein Eng Des Sel (2012) 25:591–601. 10.1093/protein/gzs042 22843678

[59] Perchiacca JM Lee CC Tessier PM. Optimal charged mutations in the complementarity-determining regions that prevent domain antibody aggregation are dependent on the antibody scaffold. Protein Eng Des Sel (2014) 27:29–39. 10.1093/protein/gzt058 24398633

[60] Chan P Warwicker J. Evidence for the adaptation of protein pH-dependence to subcellular pH. BMC Biol (2009) 7:69. 10.1186/1741-7007-7-69 19849832 PMC2770037

[61] Barthelemy PA Raab H Appleton BA Bond CJ Wu P Wiesmann C Comprehensive analysis of the factors contributing to the stability and solubility of autonomous human VH domains. J Biol Chem (2008) 283:3639–54. 10.1074/jbc.M708536200 18045863

