# Supplementary material for: Stability-Diversity Tradeoffs Impose Fundamental Constraints on Selection of Synthetic Human VH/VL Single-Domain Antibodies from In Vitro Display Libraries
Source: Front Immunol. 2017 Dec 12;8:1759. doi: 10.3389/fimmu.2017.01759 (PMC5763143; doi:10.3389/fimmu.2017.01759)
Supplement: Supplementary file 1 [file data_sheet_1.pdf]

Supplementary Material

Stability-diversity tradeoffs impose fundamental constraints on selection of synthetic human V_H/V_L single-domain antibodies from *in vitro* display libraries

Kevin A. Henry[1], Dae Young Kim[1], Hiba Kandalaft[1], Michael J. Lowden[1], Qingling Yang[1], Joseph D. Schrag[2], Greg Hussack[1], C. Roger MacKenzie[1,3], and Jamshid Tanha[1,3,4*]

[1]Human Health Therapeutics Research Centre, National Research Council Canada, 100 Sussex Drive, Ottawa, Ontario, Canada, K1A 0R6

[2]Human Health Therapeutics Research Centre, National Research Council Canada, 6100 Royalmount Avenue, Montréal, Québec, Canada, H4P 2R2

[3]School of Environmental Sciences, University of Guelph, 50 Stone Road East, Guelph, Ontario, Canada, N1G 2W1

[4]Department of Biochemistry, Microbiology and Immunology, University of Ottawa, 451 Smyth Road, Ottawa, Ontario, Canada, K1H 8M5

*Address correspondence to:

Jamshid Tanha, Ph.D.

Human Health Therapeutics Portfolio, National Research Council Canada,

100 Sussex Drive, Ottawa, ON, Canada, K1A 0R6

Tel +1-613-990-7206 or Fax +1-613-952-9092

jamshid.tanha@nrc-cnrc.gc.ca

	FR1	CDR-H1 (H31-H35B)	FR2	CDR-H2 (H50-65)	FR3	CDR-H3 (H95-102)	FR4
	1 2 3		4	5 6	7 8 9	10	11
	123456789012345678901234567890	12345	67890123456789	012abc3456789012345	67890123456789012abc345678901234	567890abcd12	34567890123
VH428	QLQLQESGGGLVQPGGSLRLSCAASGFTFS	SYAMS	WFRQAPGKGLEWVG	FIRSKAYGGTTEYAASVKG	RFTISRDDSKSIAYLQMNSLRAEDTAMYYCAR	RAKDGYNSPEDY	WGQGTLVTVSS
VH428$_{ss}$	QLQLQESGGGLVQPGGSLRLSCAASGFTFS	SYAMS	WFRQAPGKGLEWVC	FIRSKAYGGTTEYAASVKG	RFTCSRDDSKSIAYLQMNSLRAEDTAMYYCAR	RAKDGYNSPEDY	WGQGTLVTVSS
VH420	QVQLVESGGGLVKPGGSLRLSCAASGFTFS	NAWMT	WVRQAPGKGLEWVG	RIKTKTDGGTTDYAAPVKG	RFTISRDDSKNTLYLQMNSLKTEDTAVYYCTT	DRDHSS----GS	WGQGTLVTVSS
VH420$_{ss}$	QVQLVESGGGLVKPGGSLRLSCAASGFTFS	NAWMT	WVRQAPGKGLEWVC	RIKTKTDGGTTDYAAPVKG	RFTCSRDDSKNTLYLQMNSLKTEDTAVYYCTT	DRDHSS----GS	WGQGTLVTVSS
VH429	EVQLVESGGTLVQPGGSLRLSCAASGFTFI	NYAMS	WVRQAPDKGLDWVS	TISN--NGGATYYADSVKG	RFTISRDNSNNTLYLQMNSLRPDDTAVYYCAK	GPINTGRY--GD	WGQGTLVTVSS
VH429$_{ss}$	EVQLVESGGTLVQPGGSLRLSCAASGFTFI	NYAMS	WVRQAPDKGLDWVC	TISN--NGGATYYADSVKG	RFTCSRDNSNNTLYLQMNSLRPDDTAVYYCAK	GPINTGRY--GD	WGQGTLVTVSS
VHB82	QVQLQESGGGLVQPGGSLRLSCAASGFTFS	SYAMS	WVRQAPGKGLEWVS	AISG--SGGSTYYADSVKG	RFTISRDNSKNTLYLQMNSLRAEDTAVYYCGT	DMEV--------	WGKGTTVTVSS
VHB82$_{ss}$	QVQLQESGGGLVQPGGSLRLSCAASGFTFS	SYAMS	WVRQAPGKGLEWVC	AISG--SGGSTYYADSVKG	RFTCSRDNSKNTLYLQMNSLRAEDTAVYYCGT	DMEV--------	WGKGTTVTVSS
VHM81	EVQLVQSGGGLVQPGRSLRLSCAASGFTFD	DYAMH	WVRQAPGKGLEWVS	GISG--SGASTYYADSVKG	RFTISRDNSKNTLYLQMNSLRAGDTALYYCAR	QSITGPTGAFDV	WGQGTMVTVSS
VHM81$_{ss}$	EVQLVQSGGGLVQPGRSLRLSCAASGFTFD	DYAMH	WVRQAPGKGLEWVC	GISG--SGASTYYADSVKG	RFTCSRDNSKNTLYLQMNSLRAGDTALYYCAR	QSITGPTGAFDV	WGQGTMVTVSS

	FR1	CDR-L1 (L24-34)	FR2	CDR-L2 (L50-56)	FR3	CDR-L3 (L89-97)	FR4
	1 2	3	4	5	6 7 8	9	10
	12345678901234567890123	4567890a1234	567890123456789	0123456	78901234567890123456789012345678	901234567	8901234567
VL382	EIVMTQSPGTLSLSPGDRATLSC	RASQDIS-TYLA	WYQQKPGQAPRLLIY	SASRRAT	GIPDRFSGSGSGTDFTLTISRLEPEDFAVYYC	QQYSSSPKT	FGQGTKVTVL
VL382$_{ss}$	EIVMTQSPGTLSLSPGDRATLSC	RASQDIS-TYLA	WYQQKPGQAPRLLCY	SASRRAT	GIPDRFSCSGSGTDFTLTISRLEPEDFAVYYC	QQYSSSPKT	FGQGTKVTVL
VL383	ETTLTQSPGTLSLSPGERATLSC	RASQSVS-KNLA	WYQKKPGQSPRLLIH	SISTRAT	GVPARFSGGGSGTDFTLTISRLEPEDFAVYYC	QQYGNSPQT	FGQGTKVEIK
VL383$_{ss}$	ETTLTQSPGTLSLSPGERATLSC	RASQSVS-KNLA	WYQKKPGQSPRLLCH	SISTRAT	GVPARFSCGGSGTDFTLTISRLEPEDFAVYYC	QQYGNSPQT	FGQGTKVEIK
VL325	EIVLTQSPTTLSLSPGERATLSC	RASQSVG-RYLA	WYQQRPGQAPRLLVF	DTSNRAP	GVPARFSGRGSGTLFTLTISSLEPEDSAVYFC	QQYGSSPRT	FGHGTKVTVL
VL325$_{ss}$	EIVLTQSPTTLSLSPGERATLSC	RASQSVG-RYLA	WYQQRPGQAPRLLCF	DTSNRAP	GVPARFSCRGSGTLFTLTISSLEPEDSAVYFC	QQYGSSPRT	FGQGTKVTVL
VL330	DIQMTQSPPSLSAFVGDRVTITC	RASESVG-NSLS	WYQLKPGKNPRLLVS	GGSFLQS	GVSARFSGSGAGTLFTLTITGLRLDDSATYYC	QQSDAVPRT	FGHGTKVSVL
VL330$_{ss}$	DIQMTQSPPSLSAFVGDRVTITC	RASESVG-NSLS	WYQLKPGKNPRLLCS	GGSFLQS	GVSARFSCSGAGTLFTLTITGLRLDDSATYYC	QQSDAVPRT	FGHGTKVSVL
VL335	EIVMTQSPATLSLSPGERATLSC	RASQSVSSSSLA	WYQQKPGQAPRLLIY	GTSNRAT	GIPDRFSGSGSGTHFTLTINRLEPGDFAVYYC	QQYGSSPRT	FGQGTKVTVL
VL335$_{ss}$	EIVMTQSPATLSLSPGERATLSC	RASQSVSSSSLA	WYQQKPGQAPRLLCY	GTSNRAT	GIPDRFSCSGSGTHFTLTINRLEPGDFAVYYC	QQYGSSPRT	FGQGTKVTVL

Supplementary Figure S1. Full-length amino acid sequences of human autonomous V_H/V_L sdAb scaffolds used in this study. Numbering is according to Kabat.

VHB82$_{SS}$ Randomization Position

	CDR-H1					CDR-H2				CDR-H3																			
	35 (X3)	36 (X2)	37 (X1)	38 (X1)	40 (X3)	57 (X1)	58 (X1)	59 (X1)	62 (X4)	107 (X1)	108 (X3)	109 (X5)	110 (X2)	111 (X2)	111a (X2)	111b (X2)	111c (X2)	111d (X2)	111e (X2)	112d (X2)	112c (X2)	112b (X2)	112a (X2)	112 (X2)	113 (X2)	114 (X2)	115 (X1)	116 (X2)	117 (X1)
*	0.13	0.11	0.07	0.13	0.04	0.36	0.23	0.23	0.51	0.03	0.41	0.11	0.30	0.34	0.35	0.37	0.42	0.44	0.46	0.41	0.43	0.47	0.45	0.42	0.42	0.44	0.40	0.76	0.49
A	8.79	6.68	10.18	9.37	7.17	5.88	7.42	8.54	6.88	8.02	8.69	14.84	5.97	6.02	6.10	5.95	5.63	5.54	5.41	5.63	5.50	5.65	5.88	6.13	6.11	6.14	8.73	5.96	7.99
C	0.05	0.06	0.06	0.09	0.08	0.16	0.15	0.17	0.18	0.03	0.17	0.27	0.13	0.15	0.15	0.16	0.18	0.15	0.18	0.21	0.18	0.18	0.17	0.17	0.18	0.18	0.25	0.27	0.26
D	16.35	10.68	22.87	23.04	18.24	29.48	24.39	24.83	14.84	19.71	12.96	0.22	11.38	11.49	11.30	11.27	11.32	11.19	11.30	11.16	11.33	11.12	11.23	11.28	11.43	11.40	23.03	12.42	26.27
E	12.82	8.91	0.03	0.02	12.97	0.07	0.08	0.09	18.57	0.12	13.45	0.19	10.24	10.16	10.04	9.72	9.48	9.32	9.04	8.93	9.28	9.46	9.64	9.93	9.85	9.72	0.08	10.82	0.07
F	0.05	2.33	3.89	4.37	0.02	3.49	3.98	4.29	0.14	4.18	0.11	0.17	3.11	3.32	3.23	3.20	3.25	3.26	3.29	3.39	3.31	3.27	3.28	3.20	3.15	3.14	4.38	2.91	4.15
G	5.00	4.88	0.10	0.06	8.21	0.76	0.12	0.17	9.81	0.11	4.97	11.25	5.37	5.10	5.14	5.19	5.15	5.19	5.30	5.33	5.27	5.20	5.32	5.44	5.42	5.40	0.48	4.89	0.47
H	9.95	8.56	12.53	12.38	12.04	12.88	14.02	13.54	0.11	14.59	10.19	0.22	8.88	8.77	8.74	8.63	8.61	8.44	8.52	8.71	8.46	8.50	8.62	8.60	8.70	8.76	13.81	8.83	14.78
I	2.48	1.95	3.52	3.96	1.82	3.18	3.87	3.75	4.68	4.38	3.07	0.23	2.42	2.40	2.47	2.50	2.54	2.50	2.56	2.55	2.55	2.52	2.47	2.38	2.29	2.34	3.89	2.64	3.58
K	2.20	2.01	0.01	0.00	2.28	0.01	0.01	0.02	5.08	0.03	3.31	0.14	2.50	2.45	2.45	2.46	2.46	2.36	2.40	2.29	2.40	2.41	2.48	2.37	2.34	2.27	0.06	2.49	0.07
L	4.86	3.40	5.20	4.87	3.95	5.55	6.66	6.16	0.19	4.93	3.83	7.12	3.17	3.14	3.18	3.27	3.40	3.44	3.54	3.60	3.52	3.46	3.39	3.27	3.33	3.33	4.62	3.19	2.97
M	0.05	0.04	0.06	0.06	0.07	0.07	0.12	0.13	0.15	0.06	0.11	0.20	0.10	0.13	0.14	0.14	0.15	0.16	0.15	0.17	0.15	0.16	0.16	0.16	0.15	0.17	0.09	0.19	0.09
N	0.04	0.05	0.06	0.06	0.06	0.05	0.07	0.06	0.06	0.35	0.11	0.16	0.13	0.14	0.15	0.15	0.17	0.17	0.16	0.14	0.17	0.17	0.17	0.18	0.16	0.16	0.24	0.09	0.27
P	6.47	5.92	8.60	7.44	5.02	7.94	8.86	8.29	0.15	8.70	7.26	11.59	5.26	5.26	5.20	5.17	5.06	4.97	5.06	4.98	5.12	5.07	5.17	5.15	5.11	5.03	7.27	4.86	6.92
Q	12.30	10.55	0.05	0.06	13.04	0.05	0.08	0.09	0.14	0.30	9.93	0.21	7.64	7.64	7.75	7.86	8.01	8.02	7.93	7.96	8.04	8.07	7.83	7.80	7.96	7.96	0.11	7.55	0.09
R	3.10	2.90	0.11	0.13	3.10	0.04	0.21	0.26	6.86	0.49	4.14	6.68	3.42	3.06	3.38	3.34	3.26	3.21	3.13	3.20	3.27	3.32	3.33	3.29	3.26	3.21	0.60	3.58	0.75
S	5.25	5.36	7.90	7.99	5.55	7.82	8.84	9.33	12.19	9.91	6.64	11.13	6.34	6.65	6.46	6.61	6.71	7.01	6.80	6.94	6.85	6.79	6.75	6.69	6.67	6.66	9.31	6.35	9.53
T	5.83	5.57	8.57	8.53	2.05	6.81	5.80	5.15	9.55	10.11	6.94	11.49	5.27	4.85	5.14	5.00	5.08	4.98	5.00	4.90	5.01	4.94	4.94	4.87	4.91	4.78	6.92	4.65	6.23
V	4.14	3.21	5.49	5.51	4.15	5.65	5.69	6.00	8.91	3.54	3.26	7.23	3.68	3.73	3.62	3.70	3.78	3.84	3.89	3.93	3.79	3.83	3.75	3.69	3.73	3.67	5.25	3.44	5.32
W	0.05	9.55	0.02	0.01	0.12	0.03	0.03	0.05	0.13	0.07	0.22	16.32	7.19	7.39	7.39	7.68	7.92	8.21	8.27	8.30	8.02	8.00	7.72	7.63	7.50	7.72	0.05	6.61	0.03
Y	0.08	7.28	10.69	11.90	0.02	9.73	9.35	8.84	0.87	10.35	0.22	0.20	7.50	7.82	7.61	7.62	7.43	7.60	7.61	7.28	7.35	7.42	7.25	7.36	7.31	7.53	10.44	7.49	9.68

VH428 Randomization Position

	CDR-H1					CDR-H2									CDR-H3																
	35 (X3)	36 (X2)	37 (X1)	38 (X1)	40 (X3)	57 (X1)	58 (X1)	59 (X1)	60 (X1)	61 (X1)	62 (X4)	107 (X1)	108 (X3)	109 (X5)	110 (X2)	111 (X2)	111a (X2)	111b (X2)	111c (X2)	111d (X2)	111e (X2)	112d (X2)	112c (X2)	112b (X2)	112a (X2)	112 (X2)	113 (X2)	114 (X2)	115 (X1)	116 (X2)	117 (X1)
*	0.13	0.12	0.09	0.17	0.04	0.43	0.26	0.30	0.34	0.38	0.90	0.04	0.73	0.14	0.45	0.47	0.50	0.49	0.51	0.48	0.52	0.56	0.57	0.57	0.58	0.52	0.52	0.56	0.49	0.91	0.62
A	8.70	7.47	9.38	9.98	7.57	10.74	10.20	9.99	10.24	9.98	10.99	6.92	6.59	12.50	6.27	5.95	6.11	5.88	5.62	5.46	5.66	5.63	5.50	5.59	5.87	6.15	6.18	6.54	8.90	6.96	8.13
C	0.05	0.07	0.06	0.11	0.09	0.29	0.21	0.20	0.24	0.27	0.33	0.04	0.28	0.45	0.19	0.21	0.22	0.22	0.24	0.25	0.23	0.25	0.23	0.24	0.24	0.24	0.23	0.25	0.33	0.39	0.35
D	15.97	11.13	22.93	23.21	18.67	26.38	23.27	23.57	22.78	23.32	19.61	21.89	14.09	0.30	10.79	11.03	11.04	10.78	10.75	10.86	10.67	10.69	10.65	10.81	10.78	10.93	11.12	11.04	22.45	11.95	25.56
E	13.09	10.18	0.04	0.05	14.15	0.09	0.09	0.07	0.53	0.16	19.06	0.32	13.12	0.23	9.91	9.63	9.70	9.46	8.81	8.79	8.68	8.41	8.64	8.86	9.23	9.67	9.40	9.85	0.10	10.97	0.08
F	0.05	2.59	4.00	4.62	0.11	3.54	3.85	3.89	3.88	4.20	0.16	4.29	0.20	0.32	3.09	3.27	3.17	3.19	3.22	3.36	3.20	3.28	3.13	3.06	3.03	3.12	2.98	3.15	4.16	2.95	3.89
G	4.64	4.56	0.10	0.07	7.96	0.48	0.14	0.11	0.13	0.46	5.99	0.14	6.07	10.74	5.67	5.47	5.50	5.50	5.33	5.43	5.51	5.65	5.87	5.57	5.66	5.80	5.90	5.83	0.52	5.38	0.53
H	9.90	8.19	12.65	11.86	12.11	12.07	12.40	12.29	12.44	12.76	0.18	14.24	10.23	0.27	8.49	8.41	8.42	8.45	8.45	8.30	8.44	8.31	8.36	8.32	8.38	8.33	8.45	8.25	13.29	8.24	14.33
I	2.57	2.35	3.72	4.10	1.10	3.21	3.59	3.63	3.64	3.66	4.52	4.63	3.06	0.33	2.30	2.38	2.36	2.55	2.51	2.70	2.51	2.61	2.52	2.49	2.37	2.30	2.25	2.35	3.73	2.64	3.47
K	2.39	2.44	0.01	0.00	2.51	0.01	0.01	0.01	0.02	0.03	5.64	0.03	3.32	0.16	2.25	2.26	2.28	2.25	2.26	2.25	2.11	2.16	2.25	2.18	2.22	2.16	2.10	2.04	0.07	2.23	0.08
L	4.67	2.87	5.33	3.89	3.79	4.70	5.08	5.28	5.14	5.32	0.20	4.28	3.43	7.25	3.20	3.28	3.25	3.36	3.71	3.67	3.83	3.80	3.70	3.69	3.42	3.40	3.33	3.19	4.62	2.93	3.90
M	0.11	0.06	0.14	0.07	0.09	0.09	0.13	0.12	0.13	0.16	0.17	0.09	0.12	0.30	0.13	0.18	0.18	0.18	0.21	0.17	0.22	0.21	0.19	0.21	0.21	0.19	0.20	0.18	0.10	0.21	0.10
N	0.06	0.08	0.08	0.08	0.10	0.06	0.11	0.11	0.11	0.12	0.11	0.53	0.13	0.24	0.17	0.20	0.19	0.20	0.19	0.20	0.20	0.19	0.20	0.21	0.21	0.21	0.20	0.20	0.29	0.13	0.33
P	6.53	6.23	7.96	8.08	5.65	8.22	8.45	8.40	8.55	8.52	0.15	8.09	6.23	11.34	5.32	5.14	5.20	5.22	5.17	5.08	5.32	5.15	5.06	5.07	5.04	5.14	4.96	5.13	7.34	5.32	6.72
Q	12.40	8.26	0.07	0.09	11.27	0.07	0.11	0.10	0.10	0.13	0.21	0.11	9.20	0.25	7.44	7.43	7.54	7.82	8.13	8.16	8.01	8.14	8.33	8.24	8.02	7.69	7.88	7.35	0.12	6.87	0.10
R	3.21	3.23	0.14	0.21	3.45	0.04	0.28	0.34	0.41	0.46	6.87	1.11	4.64	6.27	3.43	3.43	3.34	3.27	3.31	3.29	3.40	3.32	3.30	3.34	3.45	3.38	3.34	3.39	0.80	3.78	0.97
S	5.18	5.29	8.04	7.67	6.34	7.20	8.01	7.95	7.92	8.01	7.39	9.14	7.13	12.70	6.46	6.57	6.49	6.68	6.70	6.72	6.82	7.02	6.82	6.69	6.61	6.44	6.52	6.30	9.04	6.13	9.48
T	6.22	6.41	8.77	7.69	0.57	7.07	8.40	8.27	8.10	6.14	9.87	8.35	6.53	11.78	5.58	5.45	5.46	5.33	5.26	5.24	4.88	5.17	5.10	5.19	5.25	5.44	5.41	5.58	7.56	5.53	6.50
V	4.01	3.28	5.59	5.41	4.26	5.65	5.12	5.20	5.14	5.52	7.17	4.82	4.26	7.65	3.81	3.84	3.74	3.79	3.83	3.79	3.74	3.97	3.78	3.87	3.79	3.68	3.79	3.75	5.39	3.35	5.46
W	0.04	6.94	0.02	0.01	0.14	0.03	0.03	0.02	0.03	0.05	0.13	0.11	0.31	16.43	7.10	7.55	7.41	7.63	8.41	8.26	8.46	8.31	8.57	8.43	8.23	7.65	7.75	7.07	0.05	5.42	0.03
Y	0.08	8.24	10.87	12.61	0.04	9.64	10.27	10.16	10.12	10.36	0.37	10.83	0.33	0.35	7.95	7.86	7.89	7.73	7.41	7.55	7.60	7.16	7.24	7.37	7.40	7.56	7.50	7.98	10.64	7.72	9.37

VL383$_{SS}$ Randomization Position

	CDR-L1			CDR-L2			CDR-L3					
	36 (X1)	37 (X1)	38 (X1)	57 (X1)	66 (X1)	68 (X1)	107 (X1)	108 (X2)	109(X2)	113(X2)	114(X2)	116(X2)
*	0.01	0.01	0.01	0.00	0.01	0.02	0.00	0.05	0.07	0.06	0.02	0.04
A	10.59	10.90	8.86	14.87	13.38	10.28	12.25	7.92	8.87	8.23	8.54	7.37
C	0.02	0.03	0.00	0.02	0.01	0.01	0.03	0.04	0.05	0.08	0.01	0.02
D	22.70	16.65	16.62	16.71	17.26	18.54	19.69	9.05	9.20	8.17	7.89	9.53
E	0.03	0.03	0.01	0.02	0.01	0.01	0.10	12.19	13.28	12.31	11.98	13.30
F	4.41	4.90	4.70	3.07	4.16	4.87	4.69	3.02	3.67	3.01	3.18	3.92
G	0.04	0.07	0.04	0.03	0.04	0.48	0.06	5.75	6.45	4.74	4.93	6.12
H	11.61	13.15	13.44	11.65	12.01	15.04	11.02	7.63	8.65	8.72	8.21	7.01
I	4.94	5.29	4.80	4.10	5.14	6.19	5.44	4.30	4.73	3.73	4.17	5.29
K	0.00	0.00	0.00	0.00	0.01	0.01	0.03	4.13	4.70	4.46	4.91	3.97
L	4.17	4.64	6.22	5.44	5.28	4.05	3.89	2.92	3.67	2.56	2.49	0.21
M	0.00	0.00	0.01	0.01	0.02	0.01	0.01	0.00	0.01	0.02	0.01	0.00
N	0.04	0.01	0.01	0.01	0.03	0.03	0.01	0.03	0.02	0.02	0.01	0.03
P	11.65	15.58	15.41	17.67	14.08	13.85	9.09	7.97	9.78	9.24	9.51	6.76
Q	0.03	0.01	0.02	0.01	0.02	0.01	0.41	5.74	7.10	5.74	6.06	4.27
R	0.01	0.03	0.02	0.02	0.35	0.01	0.02	3.37	3.78	2.67	3.15	2.30
S	8.63	7.49	8.17	6.97	7.21	8.81	10.43	5.01	6.54	5.76	5.14	6.28
T	7.06	7.94	8.52	7.94	8.20	2.49	6.85	5.72	6.21	5.05	3.76	5.45
V	4.67	4.47	3.64	3.59	4.42	4.01	3.84	2.38	3.21	3.16	2.38	2.85
W	0.00	0.00	0.00	0.00	0.00	0.00	0.02	5.62	6.40	5.05	6.48	6.19
Y	9.36	8.77	9.48	7.84	8.35	11.27	12.12	7.15	8.87	7.21	7.17	9.09

Supplementary Figure S2. Amino acid representation at each randomization position in the VHB82$_{SS}$, VH428 and VL383$_{SS}$ libraries. Asterisks denote stop codons.

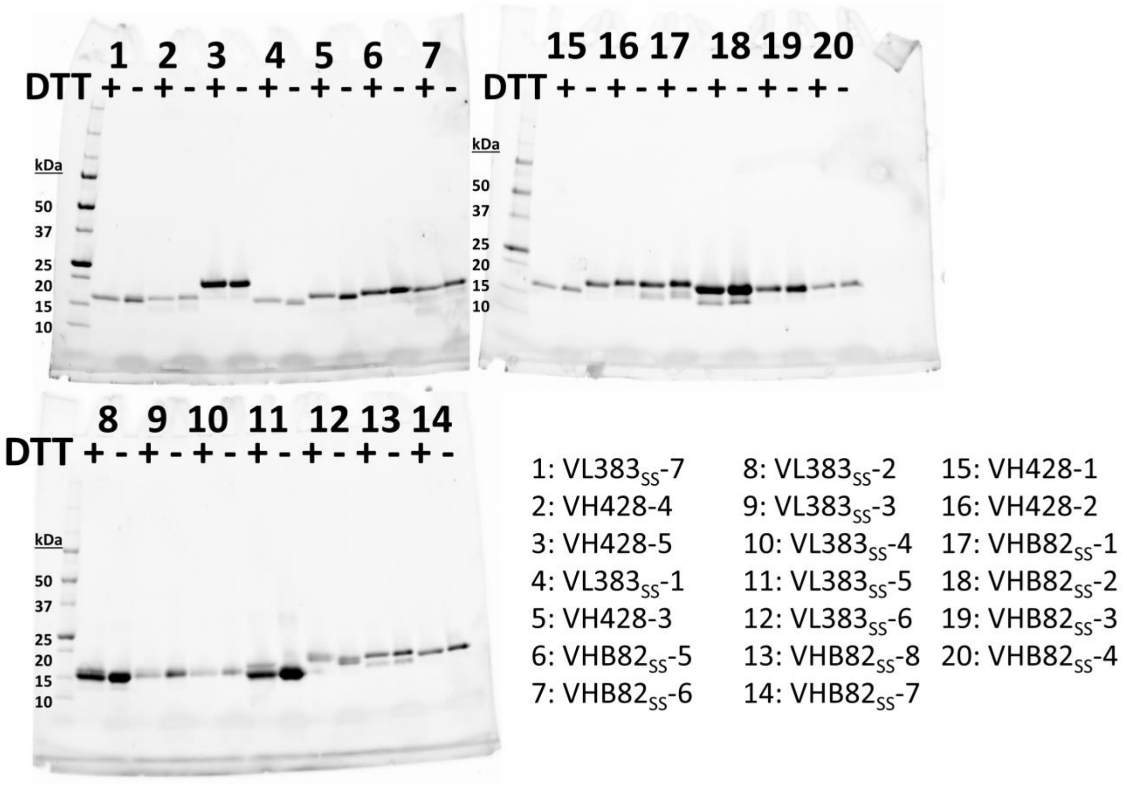

Supplementary Figure S3. SDS-PAGE in reducing and non-reducing conditions of all antigen-specific sdAbs isolated from the VL383$_{SS}$, VH428 and VHB82$_{SS}$ synthetic human V_H/V_L sdAb libraries. 1-5 µg of each IMAC-purified sdAb was electrophoresed in a Mini-PROTEAN® TGX stain-free gel (Bio-Rad); the loading buffer for the samples contained 40 mM Tris, pH 6.8, 6% (v/v) glycerol, 2% (w/v) SDS and 0.05% (w/v) bromophenol blue. DTT was added to some samples at 15 mM (+ DTT). Molecular weight marker is Bio-Rad Precision Plus Protein™ Unstained Protein Standards, Cat. #1610363.

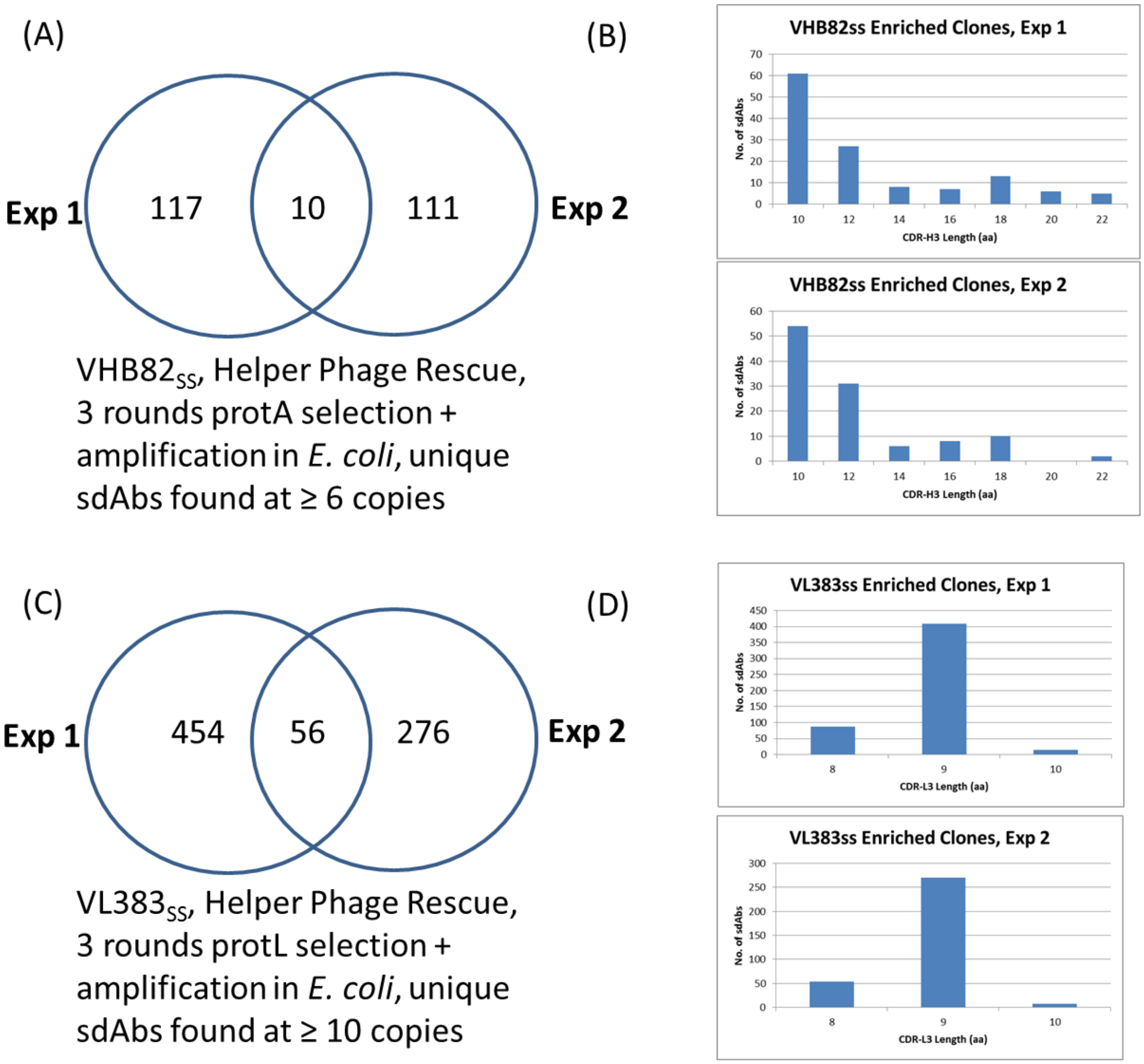

Supplementary Figure S4. Properties of human V_H and V_L sdAbs enriched by stability selection. (A) Overlap of unique VH sdAbs enriched from the $VHB82_{SS}$ library by replicate stability selection. (B) CDR-H3 length distribution of unique VH sdAbs enriched from the $VHB82_{SS}$ library by replicate stability selection. (C) Overlap of unique VL sdAbs enriched from the $VL383_{SS}$ library by replicate stability selection. (D) CDR-L3 length distribution of unique VL sdAbs enriched from the $VL383_{SS}$ library by replicate stability selection.

VL383$_{SS}$ Randomization Position, Helper Phage Rescue, 3 Rounds of Stability Selection, REPLICATE 1												
	CDR-L1			CDR-L2			CDR-L3					
	36 (X1)	37 (X1)	38 (X1)	57 (X1)	66 (X1)	68 (X1)	107 (X1)	108 (X2)	109(X2)	113(X2)	114(X2)	116(X2)
*	0.6	1.6	1.2	0.0	0.0	0.0	0.0	0.0	0.0	0.0	11.4	0.0
A	16.3	5.9	9.6	35.3	10.8	13.1	3.9	14.5	13.2	7.1	0.0	9.0
C	0.0	0.0	0.0	0.0	0.0	0.0	0.0	0.0	0.0	0.0	0.0	0.0
D	44.1	28.8	25.5	7.3	15.7	24.3	12.2	7.5	10.6	14.3	5.5	2.5
E	0.0	0.0	0.0	0.0	0.0	0.0	0.2	10.6	12.8	7.1	17.6	8.8
F	1.2	0.2	0.6	0.4	1.0	0.6	2.7	2.5	4.5	14.3	2.2	2.4
G	0.0	0.0	0.0	0.0	0.2	3.5	0.4	4.5	2.8	14.3	5.5	3.9
H	10.2	21.0	26.1	1.0	19.8	17.5	11.6	12.2	7.1	0.0	8.0	10.4
I	0.2	1.0	0.0	6.7	12.7	1.6	1.2	1.0	1.9	0.0	1.8	1.4
K	0.0	0.0	0.0	0.0	0.0	0.0	0.2	5.9	2.4	0.0	5.3	2.9
L	1.0	0.0	5.9	0.6	6.7	1.8	0.8	1.0	0.9	7.1	2.0	0.0
M	0.0	0.0	0.0	0.0	0.0	0.0	0.0	0.0	0.0	0.0	0.0	0.0
N	0.0	0.0	0.2	0.0	0.0	0.0	0.0	0.0	0.0	0.0	0.0	0.0
P	4.3	6.5	1.4	16.9	0.4	22.2	3.7	0.8	1.2	14.3	6.5	11.4
Q	0.0	0.0	0.0	0.0	0.0	0.0	0.8	8.8	6.6	7.1	5.7	2.4
R	0.0	0.2	0.0	0.0	3.5	0.0	0.0	3.9	1.9	0.0	2.9	0.4
S	11.4	12.4	19.2	18.6	7.1	9.4	16.7	6.3	10.4	0.0	3.9	9.8
T	5.3	18.8	2.9	6.3	11.8	2.0	8.8	2.5	9.0	7.1	4.7	3.3
V	0.8	1.0	0.0	6.5	6.1	1.2	1.6	0.6	1.9	0.0	2.7	5.3
W	0.0	0.0	0.0	0.0	0.0	0.0	0.0	2.4	1.9	0.0	4.7	5.5
Y	4.7	2.7	7.5	0.6	4.3	2.9	35.3	15.1	10.9	7.1	9.6	20.6

VL383$_{SS}$ Randomization Position, Helper Phage Rescue, 3 Rounds of Stability Selection, REPLICATE 2												
	CDR-L1			CDR-L2			CDR-L3					
	36 (X1)	37 (X1)	38 (X1)	57 (X1)	66 (X1)	68 (X1)	107 (X1)	108 (X2)	109(X2)	113(X2)	114(X2)	116(X2)
*	0.3	1.8	0.9	0.0	0.3	0.0	0.0	0.0	0.0	0.0	0.0	0.0
A	15.4	8.1	9.9	38.9	11.4	14.5	4.5	15.7	10.8	12.5	10.2	9.3
C	0.0	0.0	0.0	0.0	0.0	0.0	0.0	0.0	0.0	0.0	0.0	0.0
D	46.4	27.4	26.5	6.9	19.0	27.1	12.3	6.0	7.9	25.0	6.0	3.6
E	0.0	0.0	0.0	0.0	0.0	0.0	0.0	11.7	18.0	12.5	17.5	7.2
F	1.8	0.3	0.9	0.3	0.6	0.6	2.7	3.0	5.0	12.5	2.4	2.1
G	0.0	0.0	0.0	0.0	0.3	3.3	0.3	3.3	3.6	0.0	3.9	4.5
H	9.6	19.0	27.4	0.3	17.8	16.6	11.1	13.9	8.3	0.0	6.3	9.0
I	0.3	1.2	0.0	6.6	10.8	1.5	1.2	0.9	2.2	0.0	2.1	1.2
K	0.0	0.0	0.0	0.0	0.0	0.0	0.3	5.7	1.1	0.0	6.9	3.3
L	0.9	0.3	4.8	0.9	8.1	1.5	0.9	1.8	0.7	12.5	2.1	0.0
M	0.0	0.0	0.0	0.0	0.0	0.0	0.0	0.0	0.0	0.0	0.0	0.0
N	0.0	0.0	0.3	0.0	0.0	0.0	0.0	0.0	0.0	0.0	0.0	0.0
P	6.3	6.6	1.5	16.0	0.9	20.2	3.3	0.3	0.7	0.0	7.5	10.8
Q	0.0	0.0	0.0	0.0	0.0	0.0	0.3	8.4	6.8	12.5	8.4	2.7
R	0.0	0.0	0.0	0.0	3.3	0.0	0.0	3.9	2.5	0.0	3.3	0.6
S	8.7	13.0	19.0	15.4	5.4	8.7	15.4	6.0	10.1	0.0	4.5	10.8
T	6.3	17.5	2.7	8.1	12.7	1.2	9.9	2.4	7.9	0.0	3.6	4.5
V	1.2	1.2	0.0	6.3	6.3	1.8	1.8	0.6	1.1	0.0	2.7	4.2
W	0.0	0.0	0.0	0.0	0.0	0.0	0.0	2.1	2.2	0.0	4.2	5.7
Y	2.7	3.6	6.0	0.3	3.0	3.0	35.8	14.2	11.2	12.5	8.1	20.2

Supplementary Figure S5. Amino acid representation at each randomization position in VL sdAb clones (n_1=510 and n_2=332) enriched from the VL383ss library by replicate stability selection. Asterisks denote stop codons.

VHB82$_{SS}$ Randomization Position, Helper Phage Rescue, 3 Rounds of Stability Selection, REPLICATE 1																												
	CDR-H1					CDR-H2				CDR-H3																		
	35 (X3)	36 (X2)	37 (X1)	38 (X1)	40 (X3)	57 (X1)	58 (X1)	59 (X1)	62 (X4)	107 (X1)	108 (X3)	109 (X5)	110 (X2)	111 (X2)	111a (X2)	111b (X2)	111c (X2)	111d (X2)	111e (X2)	112d (X2)	112c (X2)	112b (X2)	112a (X2)	112 (X2)	113 (X2)	114 (X2)	115 (X1)	116 (X2)	117 (X1)
*	0.0	3.1	0.8	0.0	3.9	0.0	0.0	0.0	0.0	0.0	0.0	0.0	0.0	0.0	0.0	0.0	0.0	0.0	0.0	0.0	0.0	0.0	0.0	0.0	0.0	0.0	0.0	0.0	0.0
A	15.0	7.1	15.7	7.9	1.6	11.0	15.0	9.4	4.7	3.9	4.7	19.7	4.5	10.3	5.1	6.5	8.3	18.2	20.0	20.0	0.0	0.0	3.2	10.6	5.5	11.8	13.4	3.1	8.7
C	0.0	0.0	0.0	0.0	0.0	0.0	0.0	0.0	0.0	0.0	0.0	0.0	0.0	0.0	0.0	0.0	0.0	0.0	0.0	0.0	0.0	0.0	0.0	0.0	0.0	0.0	0.0	0.0	0.0
D	11.8	16.5	15.7	26.8	5.5	52.8	18.1	24.4	23.6	17.3	16.5	0.8	21.2	15.4	17.9	12.9	4.2	9.1	0.0	0.0	0.0	16.7	22.6	15.2	15.7	14.2	24.4	21.3	26.8
E	9.4	13.4	0.0	0.0	7.1	0.0	0.0	0.0	8.7	0.8	8.7	0.0	6.1	7.7	10.3	16.1	4.2	9.1	20.0	0.0	18.2	4.2	12.9	12.1	12.6	13.4	0.0	15.7	0.0
F	0.0	0.0	1.6	1.6	0.0	1.6	0.0	0.8	0.0	2.4	0.0	0.0	1.5	0.0	2.6	3.2	0.0	0.0	0.0	0.0	0.0	8.3	0.0	4.5	3.1	0.8	5.5	1.6	1.6
G	9.4	7.1	0.0	0.0	6.3	0.0	0.0	0.0	11.0	0.0	6.3	18.1	6.1	2.6	5.1	6.5	20.8	0.0	0.0	20.0	9.1	8.3	3.2	7.6	8.7	12.6	0.0	5.5	0.0
H	7.9	11.0	25.2	18.9	28.3	12.6	15.7	23.6	0.0	18.1	12.6	0.0	12.1	10.3	12.8	3.2	8.3	0.0	0.0	0.0	0.0	8.3	6.5	9.1	12.6	7.1	15.0	7.1	11.0
I	0.8	0.0	0.8	0.8	0.8	0.0	1.6	4.7	2.4	5.5	3.1	0.0	1.5	0.0	0.0	0.0	0.0	0.0	0.0	20.0	0.0	0.0	0.0	1.5	1.6	1.6	3.1	3.9	5.5
K	4.7	2.4	0.0	1.6	2.4	0.0	0.0	0.0	5.5	0.0	9.4	0.0	3.0	5.1	0.0	0.0	0.0	0.0	0.0	20.0	9.1	0.0	0.0	4.5	1.6	4.7	0.0	2.4	0.0
L	1.6	0.8	1.6	0.8	2.4	1.6	0.8	4.7	0.0	1.6	2.4	1.6	4.5	0.0	2.6	6.5	4.2	9.1	20.0	0.0	0.0	0.0	0.0	3.0	2.4	1.6	1.6	1.6	3.1
M	0.0	0.0	0.0	0.0	0.0	0.0	0.0	0.0	0.0	0.0	0.0	0.0	0.0	0.0	0.0	0.0	0.0	0.0	0.0	0.0	0.0	0.0	0.0	0.0	0.0	0.0	0.0	0.0	0.0
N	0.0	0.0	0.0	0.0	0.0	0.0	0.0	0.0	0.0	0.0	0.0	0.0	0.0	0.0	0.0	0.0	0.0	0.0	0.0	0.0	0.0	0.0	0.0	0.0	0.0	0.0	0.0	0.0	0.0
P	15.7	4.7	7.1	8.7	0.0	3.1	26.0	7.9	0.0	14.2	7.1	12.6	1.5	5.1	7.7	9.7	12.5	27.3	20.0	0.0	9.1	12.5	6.5	1.5	4.7	5.5	10.2	1.6	8.7
Q	7.1	13.4	0.0	0.0	27.6	0.0	0.0	0.0	0.0	0.0	10.2	0.8	4.5	10.3	7.7	19.4	12.5	0.0	20.0	20.0	0.0	12.5	16.1	10.6	6.3	10.2	0.0	15.7	0.0
R	3.1	3.1	0.0	0.0	3.1	0.0	0.0	0.0	9.4	0.0	3.9	4.7	3.0	2.6	5.1	0.0	4.2	18.2	0.0	0.0	0.0	8.3	3.2	4.5	3.1	0.8	0.0	2.4	0.0
S	6.3	2.4	11.8	7.9	7.1	11.8	12.6	4.7	15.0	12.6	3.1	18.1	3.0	12.8	5.1	6.5	8.3	0.0	0.0	0.0	18.2	4.2	3.2	4.5	6.3	5.5	9.4	3.1	6.3
T	3.9	8.7	9.4	14.2	0.8	3.1	6.3	5.5	15.7	5.5	9.4	9.4	9.1	7.7	5.1	6.5	8.3	0.0	0.0	0.0	9.1	12.5	12.9	4.5	6.3	4.7	6.3	7.9	10.2
V	3.1	1.6	4.7	5.5	2.4	1.6	0.8	3.1	3.9	5.5	2.4	5.5	4.5	2.6	0.0	0.0	0.0	0.0	0.0	0.0	9.1	4.2	3.2	0.0	2.4	0.0	1.6	0.8	9.4
W	0.0	1.6	0.0	0.0	0.8	0.0	0.0	0.0	0.0	0.0	0.0	8.7	6.1	0.0	5.1	3.2	4.2	0.0	0.0	0.0	0.0	0.0	6.5	4.5	1.6	1.6	0.0	2.4	0.0
Y	0.0	3.1	5.5	5.5	0.0	0.8	3.1	11.0	0.0	12.6	0.0	0.0	7.6	7.7	7.7	0.0	0.0	9.1	0.0	0.0	18.2	0.0	0.0	1.5	5.5	3.9	9.4	3.9	8.7

VHB82$_{SS}$ Randomization Position, Helper Phage Rescue, 3 Rounds of Stability Selection, REPLICATE 2																												
	CDR-H1					CDR-H2				CDR-H3																		
	35 (X3)	36 (X2)	37 (X1)	38 (X1)	40 (X3)	57 (X1)	58 (X1)	59 (X1)	62 (X4)	107 (X1)	108 (X3)	109 (X5)	110 (X2)	111 (X2)	111a (X2)	111b (X2)	111c (X2)	111d (X2)	111e (X2)	112d (X2)	112c (X2)	112b (X2)	112a (X2)	112 (X2)	113 (X2)	114 (X2)	115 (X1)	116 (X2)	117 (X1)
*	0.0	2.4	0.8	0.0	2.4	0.0	0.0	0.0	0.0	0.0	0.0	0.0	0.0	0.0	0.0	0.0	0.0	0.0	0.0	0.0	0.0	0.0	0.0	0.0	0.0	0.0	0.0	0.0	0.0
A	9.4	7.1	13.4	8.7	3.1	9.4	7.9	6.3	5.5	11.8	6.3	17.3	6.1	7.7	5.1	0.0	0.0	0.0	20.0	20.0	0.0	0.0	6.5	10.6	1.6	5.5	12.6	5.5	7.9
C	0.0	0.0	0.0	0.0	0.0	0.0	0.0	0.0	0.0	0.0	0.0	0.0	0.0	0.0	0.0	0.0	0.0	0.0	0.0	0.0	0.0	0.0	0.0	0.0	0.0	0.0	0.0	0.0	0.0
D	11.0	13.4	7.1	26.8	2.4	43.3	15.0	21.3	15.0	15.0	14.2	0.0	15.2	10.3	2.6	12.9	0.0	0.0	0.0	0.0	0.0	8.3	12.9	9.1	9.4	9.4	25.2	15.0	18.1
E	7.1	8.7	0.0	0.0	7.9	0.0	0.0	0.0	7.1	1.6	7.1	0.0	6.1	5.1	5.1	0.0	0.0	0.0	20.0	0.0	9.1	4.2	3.2	12.1	8.7	11.8	0.0	15.7	0.0
F	0.0	0.0	0.8	0.8	0.0	1.6	0.0	0.8	0.0	3.1	0.0	0.0	0.0	2.6	2.6	3.2	0.0	0.0	0.0	0.0	0.0	0.0	3.2	0.0	2.4	0.8	3.1	0.8	0.8
G	7.1	8.7	0.0	0.0	4.7	0.0	0.0	0.0	5.5	0.0	3.1	11.8	7.6	7.7	0.0	3.2	12.5	0.0	0.0	0.0	0.0	0.0	0.0	7.6	9.4	8.7	0.0	3.9	0.0
H	6.3	7.9	20.5	15.0	17.3	10.2	15.7	22.0	0.0	11.0	11.0	0.0	3.0	2.6	5.1	9.7	12.5	0.0	0.0	0.0	0.0	0.0	0.0	9.1	11.8	7.9	10.2	5.5	15.0
I	0.0	0.0	0.8	0.8	1.6	0.0	1.6	1.6	0.8	1.6	1.6	0.8	0.0	0.0	0.0	0.0	0.0	0.0	0.0	20.0	0.0	0.0	0.0	1.5	0.8	0.8	3.9	2.4	3.1
K	3.9	0.8	0.0	0.0	1.6	0.0	0.0	0.0	7.1	0.0	6.3	0.0	1.5	0.0	5.1	3.2	0.0	0.0	0.0	0.0	0.0	4.2	0.0	3.0	1.6	3.1	0.0	2.4	0.0
L	1.6	0.0	2.4	1.6	3.1	0.0	0.8	3.1	0.0	5.5	6.3	3.1	1.5	5.1	0.0	3.2	0.0	0.0	0.0	0.0	0.0	0.0	3.2	3.0	2.4	1.6	0.8	1.6	2.4
M	0.0	0.0	0.0	0.0	0.0	0.0	0.0	0.0	0.0	0.0	0.0	0.0	0.0	0.0	0.0	0.0	0.0	0.0	0.0	0.0	0.0	0.0	0.0	0.0	0.0	0.0	0.0	0.0	0.0
N	0.0	0.0	0.0	0.0	0.0	0.0	0.0	0.0	0.0	0.0	0.0	0.0	0.0	0.0	0.0	0.0	0.0	0.0	0.0	0.0	0.0	0.0	0.0	0.0	0.0	0.0	0.0	0.0	0.0
P	11.0	5.5	8.7	5.5	0.0	4.7	23.6	5.5	0.0	9.4	3.9	9.4	1.5	0.0	10.3	3.2	0.0	18.2	0.0	0.0	0.0	8.3	3.2	6.1	6.3	4.7	7.9	3.1	7.9
Q	15.0	11.0	0.0	0.0	32.3	0.0	0.0	0.0	0.0	0.0	8.7	0.0	7.6	5.1	0.0	9.7	16.7	0.0	0.0	0.0	0.0	0.0	12.9	6.1	8.7	9.4	0.0	11.8	0.0
R	3.1	4.7	0.0	0.0	1.6	0.0	0.0	0.0	5.5	0.0	4.7	7.1	1.5	2.6	0.0	0.0	4.2	0.0	0.0	0.0	0.0	8.3	6.5	6.1	3.9	5.5	0.0	1.6	0.0
S	5.5	6.3	8.7	4.7	6.3	13.4	11.0	8.7	18.9	11.8	3.1	14.2	9.1	7.7	7.7	6.5	0.0	0.0	0.0	0.0	0.0	8.3	0.0	4.5	4.7	8.7	7.1	3.1	8.7
T	3.1	7.9	13.4	11.0	0.0	3.9	7.9	5.5	18.9	8.7	9.4	9.4	9.1	7.7	7.7	6.5	4.2	0.0	0.0	0.0	9.1	4.2	9.7	1.5	3.9	3.9	6.3	3.1	10.2
V	3.1	0.8	3.9	7.1	3.1	0.8	2.4	3.1	3.1	4.7	1.6	7.9	7.6	0.0	5.1	0.0	0.0	0.0	0.0	0.0	0.0	4.2	0.0	1.5	3.1	1.6	5.5	1.6	5.5
W	0.0	0.0	0.0	0.0	0.0	0.0	0.0	0.0	0.0	0.0	0.0	6.3	4.5	2.6	5.1	3.2	0.0	0.0	0.0	0.0	0.0	0.0	3.2	3.0	3.1	1.6	0.0	4.7	0.0
Y	0.0	2.4	7.1	5.5	0.0	0.0	1.6	9.4	0.0	3.1	0.0	0.0	4.5	0.0	5.1	0.0	0.0	0.0	0.0	0.0	0.0	0.0	0.0	1.5	5.5	2.4	4.7	5.5	7.9

Supplementary Figure S6. Amino acid representation at each randomization position in VH sdAb clones (n_1=127 and n_2=121) enriched from the VHB82ss library by replicate stability selection. Asterisks denote stop codons.

VL383SS Randomization Position, Helper Phage Rescue, 3 Rounds of Stability Selection, REPLICATE 1												
	CDR-L1			CDR-L2			CDR-L3					
	**36 (X1)**	**37 (X1)**	**38 (X1)**	**57 (X1)**	**66 (X1)**	**68 (X1)**	**107 (X1)**	**108 (X2)**	**109(X2)**	**113(X2)**	**114(X2)**	**116(X2)**
*	0.4	1.0	0.5	0.1	0.1	0.0	0.0	0.0	0.0	0.0	0.0	0.0
A	18.6	8.0	10.9	34.2	10.4	12.8	7.3	12.8	9.7	10.3	9.4	9.5
C	0.0	0.0	0.0	0.1	0.0	0.0	0.0	0.0	0.0	0.0	0.0	0.0
D	37.3	25.8	22.2	9.5	19.9	27.0	16.0	8.4	10.1	9.0	6.5	4.2
E	0.0	0.0	0.0	0.0	0.0	0.0	0.3	11.5	14.3	12.7	16.7	10.0
F	1.4	0.6	1.0	0.8	1.5	1.6	3.3	2.8	3.8	3.7	2.9	3.7
G	0.3	0.1	0.1	0.0	0.1	1.7	0.2	5.8	4.1	8.4	4.9	5.2
H	11.0	18.4	25.2	2.4	16.1	13.5	9.8	11.5	7.6	6.6	7.1	7.8
I	1.1	0.9	0.3	6.8	8.9	3.1	1.8	2.2	2.7	3.1	3.0	3.3
K	0.0	0.0	0.0	0.0	0.0	0.0	0.1	4.8	2.6	3.6	5.7	3.0
L	1.2	0.7	5.1	1.9	9.4	2.4	2.2	2.2	1.4	2.9	1.9	0.2
M	0.0	0.0	0.0	0.0	0.0	0.0	0.0	0.0	0.0	0.0	0.0	0.0
N	0.0	0.0	0.1	0.0	0.0	0.0	0.0	0.0	0.0	0.0	0.0	0.0
P	6.0	6.7	3.3	17.2	3.2	18.9	6.1	2.7	3.3	8.4	7.6	9.9
Q	0.0	0.0	0.0	0.0	0.0	0.0	0.6	6.9	6.4	6.5	6.3	3.3
R	0.0	0.1	0.1	0.0	1.7	0.1	0.1	3.0	2.4	2.8	3.1	1.5
S	10.4	12.3	19.0	12.1	6.5	9.7	13.7	5.7	8.5	5.2	4.7	8.2
T	4.7	20.0	4.9	6.6	11.6	1.9	8.5	3.7	9.7	4.6	4.7	5.3
V	2.2	1.3	0.4	6.4	5.8	2.2	1.8	1.4	1.7	2.1	2.1	3.9
W	0.0	0.0	0.0	0.0	0.0	0.0	0.1	3.6	3.0	4.3	4.5	6.0
Y	5.2	3.9	6.9	1.8	4.7	4.9	28.1	10.9	8.4	5.8	8.9	14.9

VL383SS Randomization Position, Helper Phage Rescue, 3 Rounds of Stability Selection, REPLICATE2												
	CDR-L1			CDR-L2			CDR-L3					
	**36 (X1)**	**37 (X1)**	**38 (X1)**	**57 (X1)**	**66 (X1)**	**68 (X1)**	**107 (X1)**	**108 (X2)**	**109(X2)**	**113(X2)**	**114(X2)**	**116(X2)**
*	0.4	0.9	0.4	0.1	0.1	0.0	0.0	0.0	0.0	0.0	0.0	0.0
A	18.0	8.2	11.5	34.1	10.6	12.8	7.4	12.5	8.8	9.7	9.0	9.4
C	0.0	0.0	0.1	0.1	0.0	0.0	0.0	0.0	0.0	0.0	0.0	0.0
D	37.9	27.0	23.3	9.5	20.4	27.5	16.4	8.3	10.5	9.0	6.7	4.4
E	0.0	0.0	0.0	0.0	0.0	0.0	0.3	11.8	14.7	12.6	16.9	10.0
F	1.4	0.6	1.1	0.9	1.6	1.7	3.5	2.8	3.6	3.1	3.0	3.8
G	0.3	0.1	0.1	0.0	0.1	1.7	0.2	6.0	4.2	8.4	4.6	5.4
H	11.2	18.4	25.0	2.6	15.4	13.0	9.5	11.5	7.7	6.7	7.1	7.5
I	1.2	0.9	0.4	6.9	8.3	3.1	1.9	2.3	2.8	3.0	3.1	3.4
K	0.0	0.0	0.0	0.0	0.0	0.0	0.1	4.8	2.6	4.0	5.5	3.1
L	1.3	0.8	4.4	2.0	9.6	2.4	2.2	2.2	1.6	3.0	2.0	0.2
M	0.0	0.0	0.0	0.0	0.0	0.0	0.0	0.0	0.0	0.0	0.0	0.0
N	0.0	0.0	0.1	0.0	0.0	0.0	0.0	0.0	0.0	0.0	0.0	0.0
P	5.7	6.8	3.3	17.4	3.5	18.4	6.1	2.9	3.4	9.0	7.8	10.0
Q	0.0	0.0	0.0	0.0	0.0	0.0	0.5	6.8	6.5	6.4	6.3	3.4
R	0.0	0.1	0.1	0.0	1.6	0.1	0.1	3.0	2.4	2.9	3.1	1.7
S	10.2	12.1	18.4	11.3	6.9	9.7	13.2	5.6	8.1	5.3	4.9	8.3
T	4.6	18.5	4.6	6.5	11.6	2.0	8.8	3.8	9.5	4.6	4.7	5.5
V	2.3	1.3	0.3	6.5	5.8	2.3	1.8	1.4	1.8	1.9	2.1	3.3
W	0.0	0.0	0.0	0.0	0.0	0.0	0.1	3.8	3.2	4.4	4.5	6.2
Y	5.3	4.1	6.9	2.0	4.6	5.1	27.9	10.5	8.5	5.8	8.6	14.3

Supplementary Figure S7. Amino acid representation at each randomization position in all VL sdAbs present after three rounds of replicate stability selection of the VL383ss library. Asterisks denote stop codons.

VHB82SS Randomization Position, Helper Phage Rescue, 3 Rounds of Stability Selection, REPLICATE 1																													
	CDR-H1					CDR-H2				CDR-H3																			
	35 (X3)	36 (X2)	37 (X1)	38 (X1)	40 (X3)	57 (X1)	58 (X1)	59 (X1)	62 (X4)	107 (X1)	108 (X3)	109 (X5)	110 (X2)	111 (X2)	111a (X2)	111b (X2)	111c (X2)	111d (X2)	111e (X2)	112d (X2)	112c (X2)	112b (X2)	112a (X2)	112 (X2)	113 (X2)	114 (X2)	115 (X1)	116 (X2)	117 (X1)
*	0.5	2	0.5	0.4	2.9	0.7	0.3	0.3	0.4	0	0.2	0.1	0.4	0.2	0.1	0.1	0.1	0.2	0.2	0.2	0.1	0.1	0.1	0.1	0.1	0.1	0.1	0.1	0
A	9.4	7.5	13.5	9.9	5.2	6.4	12.1	10.9	6.1	9.9	9.4	16.7	19.4	6.3	6.3	6.3	6	5.7	6	7	6.2	5.7	6	6.7	6.5	6.7	9.8	6	9
C	0	0	0.1	0.1	0	0.2	0.2	0.1	0.1	0	0.1	0.1	0.2	0.1	0.1	0.1	0.1	0	0.1	0.1	0.1	0	0	0.1	0	0	0.1	0.1	0
D	13.9	14.3	16.4	26.4	7.7	47.7	22.6	23	22.6	18.5	13.7	0.1	38.1	12.3	12.7	12.6	12.1	12.2	12.4	11.9	12	12.7	12.6	12.7	12.8	12.9	25.3	13.1	25
E	14.3	10.8	0	0	12.7	0	0.1	0.1	13.9	0.1	13.1	0.1	30.4	10.6	10.7	10.5	10.3	9.8	10.2	9.3	10.7	9.7	10.4	10.7	10.7	10.8	0	12.7	0
F	0	0.9	2.1	2.3	0	1.2	1.4	3.1	0.1	3.9	0	0.1	8.1	2.7	2.6	2.8	2.5	2.6	2.7	3	2.6	2.9	2.5	2.5	2.6	2.4	4.1	2.1	3.8
G	5.3	6.9	0.1	0.1	7.8	0.2	0.2	0.3	9.6	0.1	5.7	14.1	18.5	6.1	5.8	6.1	6.7	6	6	6.3	6	6.3	6.2	6.5	6.8	6.9	0.1	6.2	0.1
H	10.3	9.5	17.3	13.2	17	11.7	16.5	17.1	0.1	15.2	9.7	0.1	26.3	9.1	8.9	8.6	9.1	8.8	8.4	8.7	9	8.3	8.3	8.5	8.6	9.1	14.2	8.9	16.5
I	1.2	0.9	1.9	2.4	2.1	1.1	2	3	2.2	3.8	2.2	0.1	5.9	1.8	1.9	1.8	2	2.1	1.9	2.3	2.1	1.9	1.9	1.8	1.7	1.6	3.3	2	3.8
K	2.4	2.2	0	0	1.6	0	0	0	5.4	0	3.8	0	8.4	2.6	2.8	2.6	2.8	2.6	2.2	2.5	2.4	2.9	2.6	2.6	2.6	2.5	0	2.6	0
L	2.2	1.5	3.2	2.4	3.8	2.5	4.5	5.1	0.1	4.7	3.1	5.9	8.6	2.8	2.6	2.8	2.7	3	2.7	3.1	3	2.6	2.6	2.5	2.6	2.6	4	2.1	2.9
M	0.1	0.1	0.1	0	0.1	0	0	0	0	0	0	0	0.1	0.1	0	0.1	0	0.1	0.1	0.1	0	0	0	0	0	0	0	0	0
N	0.1	0.1	0.1	0.1	0.1	0.1	0.1	0.1	0.1	0.2	0.1	0.1	0.2	0.1	0.1	0.1	0.1	0	0	0.1	0.1	0.1	0.1	0.1	0	0.1	0.1	0	0.1
P	9.4	5.4	8.2	9.8	0.4	7.1	13.9	8.2	0.1	8.5	7.3	12.5	16.8	5.6	5.7	5.5	5.3	6.2	5.3	5.8	5.4	5.9	5.8	5.6	5.6	5.3	8.1	5	6.3
Q	14.1	12.9	0.1	0.1	22.5	0	0.1	0.1	0.1	0.1	10.6	0.1	25.7	8.5	8.8	9.1	9.3	9	9	8.8	8.6	9	8.9	9.3	9.2	9.3	0	9.8	0
R	3.5	3.5	0.2	0.2	2.5	0	0.2	0.2	6.1	0.3	4.5	7.5	10.5	3.1	3.4	3.1	3	3	3.4	3.2	3.2	3.3	3.2	3.1	3.2	3.1	0.1	3.2	0.1
S	5.3	6.5	10.5	7.8	5	10.1	11.3	10.5	14.6	10.9	6.7	12.4	19.3	6.8	6.6	6.7	6.6	7.1	6.6	7.1	6.8	7.1	7.1	7	6.7	6.7	9.7	6	9.2
T	5.5	5.5	11.8	10.3	2.2	4.9	6.2	4.9	13.4	10.3	7.1	11.6	15.8	4.9	5.1	5	5.1	4.7	5.8	4.7	5.1	5.3	5.1	4.8	5	4.6	6.9	4.7	7
V	2.5	1.8	4.7	4.5	6.5	2.5	4	5.5	4.8	3.6	2.5	6	9.6	3.2	3.1	3.1	3.3	3.5	3.8	3	3.1	3.4	3.5	3.1	3	3	5	3.1	6.1
W	0	3.2	0	0	0	0	0	0	0.1	0	0.1	12.3	18.2	6.5	6.3	6.5	6.4	6.9	7.5	7.2	6.5	6.4	6.6	6.2	6.1	6	0	5.7	0
Y	0	4.5	9.3	10	0	3.5	4.3	7.4	0.3	9.9	0.1	0.1	19.6	6.8	6.5	6.6	6.5	6.5	5.8	5.8	7	6.5	6.4	6.1	6.2	6.4	9.1	6.4	10

VHB82SS Randomization Position, Helper Phage Rescue, 3 Rounds of Stability Selection, REPLICATE 2																													
	CDR-H1					CDR-H2				CDR-H3																			
	35 (X3)	36 (X2)	37 (X1)	38 (X1)	40 (X3)	57 (X1)	58 (X1)	59 (X1)	62 (X4)	107 (X1)	108 (X3)	109 (X5)	110 (X2)	111 (X2)	111a (X2)	111b (X2)	111c (X2)	111d (X2)	111e (X2)	112d (X2)	112c (X2)	112b (X2)	112a (X2)	112 (X2)	113 (X2)	114 (X2)	115 (X1)	116 (X2)	117 (X1)
*	0.5	1.7	0.5	0.4	2.4	0.7	0.4	0.3	0.4	0	0.2	0.1	0.1	0.1	0.1	0.1	0.1	0.1	0.1	0.1	0.1	0.1	0.1	0.1	0.1	0.1	0.1	0.1	0.1
A	9.2	7.6	13.4	9.9	5.4	6.1	12	10.8	6.1	9.8	9.1	16.3	6.5	6.6	6.5	6.1	5.8	6	6.2	6.4	5.6	6	6.1	6.7	6.4	6.5	9.5	6.4	9
C	0	0	0.1	0.1	0	0.2	0.2	0.1	0.2	0	0.1	0.1	0.1	0	0.1	0	0.1	0.1	0	0	0	0.1	0	0	0	0	0	0.1	0
D	14.5	14.5	17.2	26.9	7.8	47.8	23.2	23.2	22.8	18.5	13.9	0.1	12.3	12.5	12.7	12.7	12.3	12.8	12.9	12	12.5	12.2	12.6	12.8	12.9	12.9	25.6	12.9	25.3
E	14.6	10.8	0	0	13	0	0.1	0.1	14	0.1	13.2	0.1	10.4	10.6	10.6	10.5	10.2	9.5	9.6	9.7	10.2	9.9	10.7	10.9	10.7	11.1	0	12.6	0
F	0	0.9	2	2.1	0.1	1.2	1.4	3	0.1	3.8	0	0.1	2.5	2.8	2.5	2.8	2.6	2.8	3	2.8	2.4	2.7	2.7	2.5	2.4	2.5	3.9	2	3.8
G	5.2	6.9	0.1	0.1	7.8	0.2	0.2	0.2	9.9	0.1	5.6	13.8	6.4	6.3	6.1	6.1	6.3	5.7	5.9	6.4	6.3	6	6.3	6.6	6.9	6.7	0.1	6.2	0.1
H	10.2	9.4	17	12.8	16.4	11.7	16.5	17	0.1	15.1	9.8	0.1	8.9	9.1	8.9	8.7	9.2	8.3	9.6	8.4	8.4	8.5	8.6	8.6	8.7	9.2	14.2	8.8	16.3
I	1.2	0.9	1.9	2.4	2.2	1.1	1.9	2.8	2.2	3.8	2.2	0.1	2	2.1	1.8	1.8	1.8	2	1.8	1.9	2	1.8	1.8	1.8	1.7	1.6	3.4	1.9	3.8
K	2.2	2.2	0	0	1.6	0	0	0	5.4	0	3.7	0.1	2.8	2.6	2.7	2.6	2.7	2.7	2.9	2.6	2.3	2.6	2.5	2.7	2.4	2.4	0	2.5	0
L	2.3	1.5	3.3	2.4	3.8	2.5	4.5	5.2	0.1	4.6	3.2	5.9	2.9	2.6	2.7	2.7	2.5	2.9	2.9	2.7	2.9	2.8	2.7	2.5	2.8	2.7	4	2.1	3
M	0.1	0.1	0.1	0	0.1	0	0.1	0	0	0	0	0.1	0.1	0	0	0	0	0	0	0.1	0.1	0	0	0	0	0	0	0	0
N	0.1	0.1	0.1	0.1	0.1	0.1	0.1	0.1	0.1	0.2	0.1	0.1	0.1	0.1	0.1	0.1	0.1	0.1	0.1	0	0.1	0.1	0.1	0.1	0.1	0	0.1	0	0.1
P	9	5.5	8.2	9.9	0.5	7.3	13.7	8.2	0.1	8.5	7.3	12.7	5.7	5.4	5.7	5.6	5.8	6	5	5.1	5.6	5.8	5.8	5.5	5.5	5.3	8.2	5	6.1
Q	14.2	13	0.1	0.1	22.4	0	0	0.1	0.1	0.1	10.3	0.1	8.5	8.6	8.8	9.3	9.2	9	9	9.4	9.4	9.5	8.9	8.8	9	9.3	0	9.7	0
R	3.4	3.3	0.2	0.2	2.5	0.1	0.2	0.3	5.9	0.3	4.4	7.5	3.5	3.1	3.3	3.3	3.2	3	3	3.1	3.1	3.3	3.1	3.3	3.2	3	0.1	3.3	0.1
S	5.2	6.4	10.5	7.7	5	9.9	11.1	10.6	14.3	10.9	6.8	12.5	6.5	6.8	6.5	6.6	7	6.5	6.9	7.5	6.7	6.9	6.8	6.8	6.7	6.6	9.5	6.1	9.2
T	5.5	5.7	11.4	10.2	2.3	4.8	6.1	5.2	13.1	10.7	7.2	12	5.5	4.8	5.2	4.9	5.1	5.3	4.5	5	5.4	5.1	5.1	4.9	5	4.5	7	4.7	6.9
V	2.5	1.9	4.5	4.6	6.7	2.6	3.8	5.4	4.9	3.5	2.6	6.1	3.3	3.1	3.1	3.2	3.2	3.7	3.9	3.1	2.9	3.6	3.3	3.1	3.1	3	5.1	3.3	6
W	0	3.2	0	0	0	0	0	0	0.1	0	0.1	12.1	5.7	6	6.1	6.5	6.5	7.1	6.8	7	7.4	6.7	6.4	6.1	6.1	6.1	0	5.7	0
Y	0	4.5	9.3	9.9	0	3.6	4.4	7.3	0.2	10	0.1	0.1	6.4	6.8	6.5	6.6	6.1	6.4	5.7	6.8	6.5	6.3	6.4	6.2	6.4	6.4	9.2	6.5	10.1

Supplementary Figure S8. Amino acid representation at each randomization position in all VH sdAbs present after three rounds of replicate stability selection of the VHB82ss library. Asterisks denote stop codons.

Supplementary Table SI. Sequences of oligonucleotide primers used in this study.

**Name**	**Sequence (5'—> 3')**	**Purpose**
M13F	TTG TAA AAC GAC GGC CAG	PCR Amplification of assembled library
M13R	GGA AAC AGC TAT GAC CAT G	PCR Amplification of assembled library
-96GIII	CCC TCA TAG TTA GCG TAA CGA TCT	Colony-PCR Sequencing
fdTGIII	GTG AAA AAA TTA TTA TTC GCA ATT CCT	Colony-PCR Sequencing
seqF-428	CGC TCT TCC GAT CTC TG NNNNN CAG CTG CAG CTG CAG GAG TCG GGG	NGS
seqF-B82S	CGC TCT TCC GAT CTC TG NNNNN CAG GTG CAG CTG CAG GAG TCG GGG	NGS
seqF-383S	CGC TCT TCC GAT CTC TG NNNNN GAA ACG ACA CTC ACG CAG TCT CCA	NGS
seqR-428	TGC TCT TCC GAT CTG AC NNNNN TGA GGA GAC GGT GAC TAG TGT TCC	NGS
seqR-B82s	TGC TCT TCC GAT CTG AC NNNNN TGA AGA GAC GGT GAC CGT GGT ACC	NGS
seqR-383S	TGC TCT TCC GAT CTG AC NNNNN TTT GAT CTC CAC CTT GGT CCC CTG	NGS

Supplementary Table SII. Expression yields from overnight *E. coli* TG1 cultures, T_ms and solubility profiles of potential human V_H sdAb scaffolds considered for use in this study.

V_H sdAb scaffold	Disulfide Linkages (IMGT numbering)	Expression Yield (mg/L)	T_m (° C)	Monomer (%)
VH44	23-104	8.2	64.2	>95
VH44$_{SS}$	23-104, 54-78	2.0	74.5	72.3
**VHB82**	**23-104**	**12.8**	**57.9**	**>95**
**VHB82$_{SS}$**	**23-104, 54-78**	**15.6**	**72.9**	**80.8**
VH419	23-104	3.4	56.9	>95
VH419$_{SS}$	23-104, 54-78	0.6	67.4	>95
**VH429**	**23-104**	**3.4**	**58.5**	**>95**
**VH429$_{SS}$**	**23-104, 54-78**	**16.0**	**71.8**	**>95**
VHM41	23-104	1.8	60.8	>95
VHM41$_{SS}$	23-104, 54-78	2.1	72.2	63.7
**VHM81**	**23-104**	**32.8**	**66.9**	**>95**
**VHM81$_{SS}$**	**23-104, 54-78**	**1.9**	**76.8**	**85.0**
**VH428**	**23-104**	**40.0**	**62.3**	**>95**
**VH428$_{SS}$**	**23-104, 54-78**	**9.6**	**73.1**	**>95**
**VH420**	**23-104**	**8.1**	**57.8**	**>95**
**VH420$_{SS}$**	**23-104, 54-78**	**33.6**	**67.3**	**>95**
VH414	23-104	11.8	55.3	>95
VH414$_{SS}$	23-104, 54-78	nd	64.7	>95
VH423	23-104	2.4	55.0	>95
VH423$_{SS}$	23-104, 54-78	nd	70.7	>95
VH413	23-104	5.8	54.2	>95
VH413$_{SS}$	23-104, 54-78	<0.1	nd	nd

nd, not determined

V_H sdAb scaffolds in bold red font were selected for CDR-shuffling experiments.

Supplementary Table SIII. Expression yields from overnight *E. coli* TG1 cultures, T_ms and solubility profiles of potential human V_L sdAb scaffolds considered for use in this study.

**V_L sdAb scaffold**	**Disulfide Linkages (IMGT numbering)**	**Expression Yield (mg/L)**	**T_m (° C)**	**Monomer (%)**
VL324	23-104	2.5 – 7.0	66.1	>95
VL324$_{SS}$	23-104, 54-78	0.5 – 1.1	73.4	>95
**VL325**	**23-104**	**34.8**	**68.5**	**>95**
**VL325$_{SS}$**	**23-104, 54-78**	**39.0**	**82.5**	**>95**
**VL335**	**23-104**	**36.4**	**61.7**	**>95**
**VL335$_{SS}$**	**23-104, 54-78**	**33.8**	**79.0**	**>95**
VL342	23-104	1.0 – 7.7	58.4	>95
VL342$_{SS}$	23-104, 54-78	1.7 - 10.8	63.8	>95
VL364	23-104	0.3 – 77.0	57.0	>95
VL364$_{SS}$	23-104, 54-78	4.7	72.3	>95
VL389	23-104	3.0 – 16.7	51.9	>95
VL389$_{SS}$	23-104, 54-78	6.5	66.3	>95
**VL383**	**23-104**	**6.8**	**57.3**	**>95**
**VL383$_{SS}$**	**23-104, 54-78**	**47.0**	**73.8**	**85.3**
**VL382**	**23-104**	**2.9**	**70.1**	**>95**
**VL382$_{SS}$**	**23-104, 54-78**	**22.8**	**83.3**	**>95**
**VL330**	**23-104**	**1.8**	**62.8**	**>95**
**VL330$_{SS}$**	**23-104, 54-78**	**16.3**	**83.7**	**>95**

nd, not determined

V_L sdAb scaffolds in bold red font were selected for CDR-shuffling experiments.
